# High triglyceride-glucose index and stress hyperglycemia ratio as predictors of adverse cardiac events in patients with coronary chronic total occlusion: a large-scale prospective cohort study

**DOI:** 10.1186/s12933-023-01883-8

**Published:** 2023-07-15

**Authors:** Yanjun Song, Kongyong Cui, Min Yang, Chenxi Song, Dong Yin, Qiuting Dong, Ying Gao, Kefei Dou

**Affiliations:** 1 Cardiometabolic Medicine Center, Department of Cardiology, Fuwai Hospital, National Center for Cardiovascular Diseases, Chinese Academy of Medical Sciences and Peking Union Medical College, Beijing, China; 2 State Key Laboratory of Cardiovascular Disease, 167, Beilishi Road, Xicheng District, Beijing, 100037 China

**Keywords:** Triglyceride-glucose index, Stress hyperglycaemia ratio, Chronic total occlusion, Coronary heart disease, Diabetes

## Abstract

**Background:**

The triglyceride-glucose (TyG) index and the stress hyperglycaemia ratio (SHR) are both positively associated with cardiovascular (CV) risk in patients with coronary heart disease. However, the prognostic value of these two biomarkers has not been well elucidated in patients with chronic total occlusion (CTO). Therefore, this study aims to evaluate the association of the TyG index and the SHR with long-term prognosis in patients with CTO.

**Methods:**

This prospective cohort study consecutively included 2740 angina patients with CTO from January 2017 to December 2018 at Fuwai Hospital. The outcomes are a composite of CV death and target vessel myocardial infarction (TVMI) and major CV cerebrovascular adverse events (MACCEs, including all-cause death, nonfatal MI, ischaemia-driven target vessel revascularization, and stroke). The association between biomarkers and prognosis was analysed by multivariable Cox proportional hazard models, and the predictive value was determined by a receiver-operating characteristic (ROC) curve.

**Results:**

During the follow-up with a median time of 3 years, 179 (6.5%) cases of MACCEs and 47 (1.7%) cases of CV death or TVMI were recorded. Patients with a high TyG index (> 9.10) and a high SHR (> 0.87) showed a significantly increased risk of CV death/TVMI (TyG index: HR 4.23, 95% CI 1.58–11.37; SHR: HR 5.14, 95% CI 1.89–13.98) and MACCEs (TyG index: HR 2.47, 95% CI 1.54–3.97; SHR: HR 2.91, 95% CI 1.84–4.60) compared with those with a low Tyg index and a low SHR (TyG < 8.56, SHR < 0.76). The area under the curve (AUC) values were 0.623 (TyG index) and 0.589 (SHR) for CV death/TVMI and 0.659 (TyG index) and 0.624 (SHR) for MACCEs. Furthermore, patients with both a high TyG index and a high SHR showed the highest risk of clinical outcomes among patients with different levels of these two biomarkers, and the AUC for the TyG-SHR combination was larger than the TyG index alone in predicting MACCE risk.

**Conclusions:**

The study revealed that a high TyG index and a high SHR were significantly correlated with poor prognosis in patients with CTO and suggested that these two biomarkers are reliable in predicting long-term prognosis in CTO patients.

**Supplementary Information:**

The online version contains supplementary material available at 10.1186/s12933-023-01883-8.

## Introduction

Chronic total occlusion (CTO) is a complex coronary lesion with a prevalence of 16–20% in patients with coronary heart disease (CHD) [1, 2]. In clinical practice, the CTO procedural success rate is not high (approximately 55–62% in Michigan in 2017), and patients with CTOs tend to have a high incidence of complications, long procedural duration, and high cost [2]. Regarding the prognosis of CTO patients, scholars reported a 10.4% 10 year cardiovascular (CV) mortality in those with successful procedures, indicating that a high CV risk existed in CTO patients [3]. Therefore, approaches identifying high-risk patients among those with CTO lesions are warranted.

The triglyceride-glucose (TyG) index was determined to be a reliable biomarker in evaluating insulin resistance [4], and the stress hyperglycaemia ratio (SHR) was suggested to precisely reflect background glucose metabolic status [5]. In previous studies, these two biomarkers have been revealed to be positively associated with the severity of coronary lesions and long-term CV risk in patients with CHD [6–8]. A high TyG index was suggested to correlate with poor prognosis in patients with CTOs in a previous retrospective study [9]. Although that was a well-conducted study, the sample size was relatively small (652 patients), which may have potentially biased the results [9]. Therefore, the prognostic value of the TyG index in CTO patients warrants investigation in a large population-based prospective cohort. Although glucose metabolism disorders were shown to be associated with a high incidence of adverse CV events in patients with CTO [10], the prognostic value of the SHR in patients with CTO lesions is still not clear.

In this large population-based prospective cohort study, we consecutively included 2740 patients with CTO lesions and conducted a follow-up with a median time of 3 years. The objective of this study was to evaluate the association of the TyG index and the SHR with long-term prognosis in CTO patients.

## Methods

### Study design and population

**Fig. 1:**
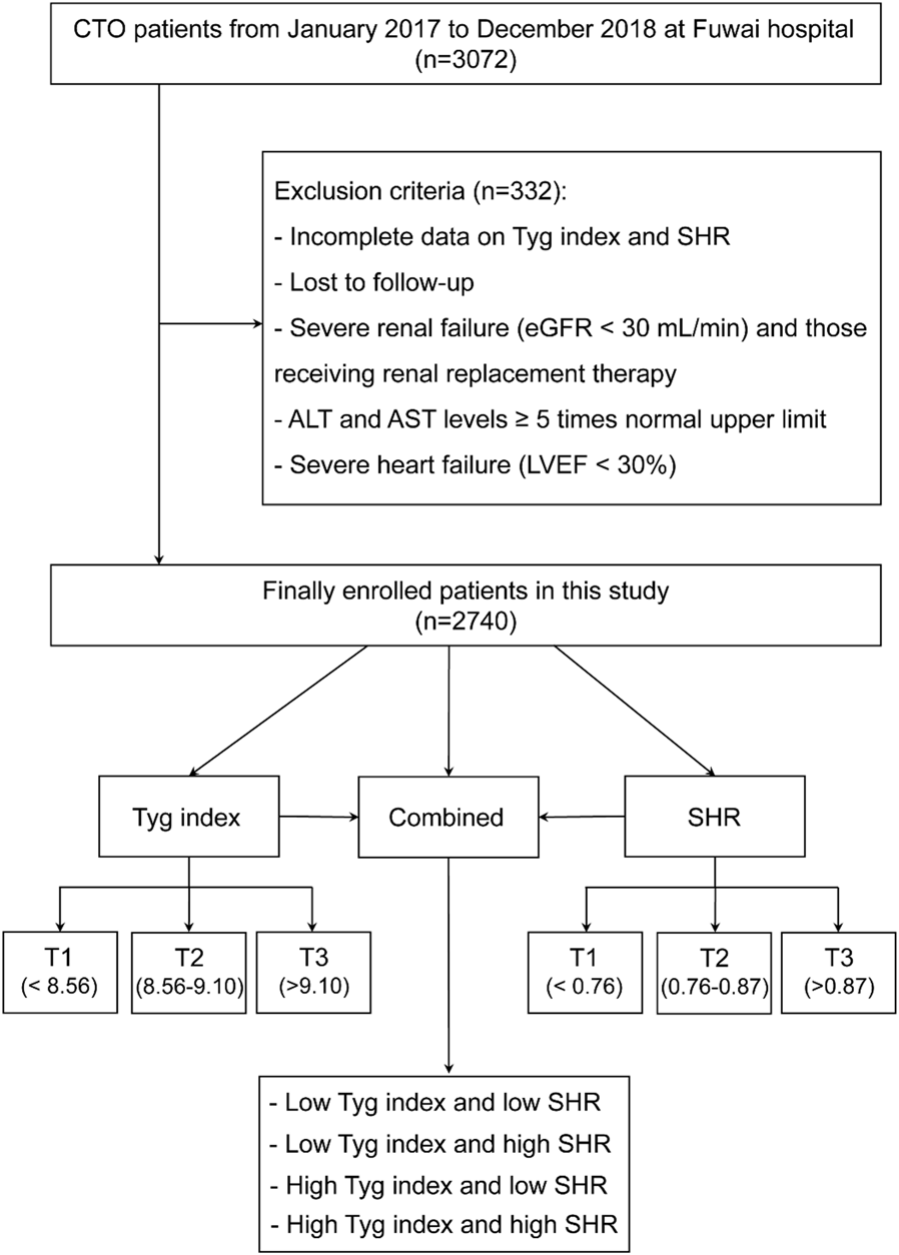
Flow chart of the enrolled patients*. CTO* chronic total occlusion, *TyG* riglyceride-glucose, *SHR* stress-hyperglycemia ratio, *ALT* alanine aminotransferase, *AST* aspartate aminotransferase, *LVEF* left ventricular ejection fraction

This was a single-centre prospective cohort study. From January 2017 to December 2018, a total of 3072 patients who underwent percutaneous coronary intervention (PCI) for CTO lesions were consecutively enrolled at FuWai Hospital, National Center for Cardiovascular Diseases. A CTO lesion was defined as a thrombolysis in myocardial infarction (TIMI) flow grade of 0 for a coronary artery with a duration of ≥ 3 months. The main exclusion criteria were incomplete data on the TyG index or SHR, severe liver and/or renal insufficiency, severe heart failure (HF), and loss to follow-up. Ultimately, a total of 2740 participants were analysed in the present study. The detailed flow chart is shown in Fig. 1.

Participants enrolled in this study were divided into different groups according to the TyG index (T1: < 8.56; T2: 8.56–9.10; T3: > 9.10) and SHR (T1: < 0.76; T2: 0.76–0.87; T3, > 0.87). A high TyG index and SHR were defined as T3, and levels of T1 and T2 were considered to be ‘low’ groups. Participants were further divided into four groups according to the combination of the TyG index and the SHR: groups of low TyG and SHR, low TyG and high SHR, high TyG and low SHR, and high TyG and SHR.

This study was conducted in compliance with the Declaration of Helsinki and was approved by the Institutional Review Board of FuWai Hospital, National Center for Cardiovascular Diseases. All participants provided written informed consent before enrolment.

### Study procedures and biochemical analysis

During hospitalization, all procedures and medical therapies were conducted according to the guideline’s recommendation and the cardiologist’s discretion. All patients received loading doses of aspirin (300 mg), clopidogrel (600 mg), or ticagrelor (180 mg) before PCI. The detailed PCI strategy was determined by experienced interventionists. All CTO lesions were first treated with the anterograde approach using dedicated coronary wires and devices; otherwise, the retrograde approach was adopted. The use of coronary wires and devices, as well as adjunctive examinations such as intravascular ultrasound and optical coherence tomography, were left to the discretion of interventionists. The procedural success of CTO-PCI was defined as residual stenosis < 30% with TIMI grade ≥ 2 antegrade flow. After patients were discharged, aspirin therapy was continued indefinitely (100 mg/day), and clopidogrel (75 mg/day) or ticagrelor (180 mg/day) was administered for ≥ 12 months.

Before coronary angiography, we obtained laboratory samples from each participant from the cubital vein after overnight fasting. All tests were conducted through the clinical chemistry department of our center. Concentrations of triglycerides, total cholesterol, low-density lipoprotein cholesterol (LDL-C), high-density lipoprotein cholesterol (HDL-C), fasting plasma glucose (FPG), and creatinine were analysed in an enzymatic assay by an automated biochemical analyser (Hitachi 7150, Tokyo, Japan). Glycosylated haemoglobin A1c (HbA1c) was measured using a Tosoh Automated Glycohemoglobin Analyser (HLC-723G8, Tokyo, Japan). Angiographic and procedural data were collected from catheter laboratory records by three experienced interventional cardiologists. Demographics, cardiovascular risk factors, clinical parameters, laboratory and imaging data, coronary angiographic and procedural details, and use of medications at discharge were prospectively collected with standardized questionnaires by independent research personnel. Notably, the TyG index was calculated using the following equation: Ln (fasting triglyceride (mmol/L) × FPG (mmol/L)/2) [11], while the SHR was calculated by the formula [FPG (mmol/L)]/[1.59 × HbA1c (%)− 2.59)] [12].

Patients who had a history of diabetes, received glucose-lowering therapy or had an FPG ≥ 7.0 mmol/L, HbA1c ≥ 6.5%, or 2 h plasma glucose ≥ 11.1 mmol/L in an oral glucose tolerance test (OGTT) were considered to have diabetes. Prediabetes was defined as FPG 5.6 ~ 6.9 mmol/L, HbA1c 5.7 ~ 6.4%, or 2 h plasma glucose 7.8 ~ 11.0 mmol/L in an OGTT according to the American Diabetes Association criteria [13]. Hypertension was defined as systolic blood pressure ≥ 140 mmHg, diastolic blood pressure ≥ 90 mmHg, or the use of antihypertensive therapy [14]. Dyslipidaemia was defined as triglycerides ≥ 150 mg/dL, total cholesterol ≥ 200 mg/dL, LDL-C ≥ 130 mg/dL or HDL-C < 40 mg/dL, or the use of cholesterol-lowering therapy [15]. Heart failure [HF] was defined as having a history of HF or left ventricular ejection fraction [LVEF] < 50% [16]. Renal dysfunction was defined as an estimated glomerular filtration rate (eGFR) < 60 mL/min/1.73 m^2^ [17].

### Follow‑up and endpoints

After discharge, participants were followed up at 6-month intervals until December 31, 2021. Data for endpoints were obtained from medical records, clinical visits, and/or telephone interviews by trained investigators who were blinded to the clinical data. The primary endpoint was the composite of CV death or target-vessel myocardial infarction (TVMI) and major adverse cardiovascular and cerebrovascular events (MACCEs), including all-cause death, nonfatal MI, ischaemia-driven target vessel revascularization, and stroke. The secondary endpoint was all-cause death, which was utilized in the survival analysis. Death was considered cardiac unless unequivocal noncardiovascular cause could be established. MI was defined as positive cardiac troponins with typical chest pain, typical electrocardiogram serial changes, identification of an intracoronary thrombus by angiography or autopsy, or imaging evidence of new loss of viable myocardium or a new regional wall-motion abnormality [18]. Ischaemia-driven lesions were defined as restenotic lesions ≥ 50% in angiography with ischaemic evidence or ≥ 70% in angiography irrespective of the ischaemic evidence. Target vessel revascularization was defined as any unplanned repeat percutaneous intervention or surgical bypass of the treated vessel with CTO lesions. Stroke was defined as a new focal neurological deficit lasting > 24 h confirmed by imaging evidence. All events were carefully verified and adjudicated by independent clinicians.

### Statistical analysis

Continuous variables were expressed as the mean ± standard deviation if conformed to the normal distribution, otherwise shown as median (interquartile range), while categorical variables were expressed as frequencies (percentages). Differences between groups were compared using one-way ANOVA, Kruskal‒Wallis H test, Pearson's chi-square test, or Fisher’s exact test, when appropriate. The correlation between the TyG index, SHR, and other continuous variables was calculated using the Spearman rank correlation test. The cumulative incidence of clinical events was estimated using Kaplan‒Meier curves, and differences were assessed with the log-rank test. Single-variable and multivariable Cox regression analyses were conducted to calculate hazard ratios (HRs) and 95% confidence intervals (CIs). In multivariable analysis, three Cox regression models were fitted. Model 1 was adjusted for age, sex, and body mass index (BMI); Model 2 was adjusted for the variables in Model 1 and smoking, diabetes, dyslipidaemia, hypertension, HF, prior MI, prior stroke, peripheral vascular disease, prior revascularization, and acute myocardial infarction; Model 3 was adjusted for the variables in Model 2 and multivessel disease, ostial lesion, bifurcation, number of lesions ≥ 2, number of stents ≥ 2, procedural success, eGFR, dual antiplatelet therapy, statin, and antidiabetic drug at discharge. The association of each co-variate with the risk of clinical outcomes with model 3 is presented in Additional file 1: Table S1, S2.

On a continuous scale, restricted cubic splines (RCSs) were used to examine the potential nonlinear relationships between the TyG index, SHR, and clinical outcomes. In addition, receiver operating characteristic (ROC) curve analysis was conducted, and areas under the curve (AUCs) were calculated to evaluate the predictive value of the TyG index, SHR, and their combination in predicting the prognosis of patients with CTO lesions. The cut-off values of the TyG index and SHR were identified by the Youden index using ROC curve analysis. The Youden Index equals the sum of sensitivity and specificity minus 1, and the optimal cut-off values refer to the TyG index and SHR that correspond to the maximum Youden Index. In addition to the primary endpoints, we conducted a survival analysis for the relationship between the TyG index, SHR, and life expectancy with the endpoint of all-cause death.

We further determined the association of the TyG index and SHR with the risk of clinical outcomes in patients with various glucose metabolism statuses (normal glucose tolerance [NGT], prediabetes, and T2DM) and procedural outcomes (success or failure). Categories were all based on the tertiles of the TyG index or SHR. Moreover, we examined the effect of the TyG index and SHR on the clinical outcomes through subgroup analysis based on important clinical variables, such as age, sex, BMI, smoking, dyslipidaemia, hypertension, HF with low LVEF (< 50%), renal dysfunction, and AMI. Sensitivity analysis was conducted by excluding patients who had clinical events within 90 days after the initial procedure. All statistical analyses were conducted with R version 3.6.0 (R Foundation for Statistical Computing, Vienna, Austria). A two-sided P value of < 0.05 was deemed statistically significant.

## Results

### Baseline characteristics according to categories of outcomes

Baseline characteristics and comparisons between patients with and without clinical outcomes are presented in Table 1. In the population enrolled in this study, the mean age was 58.5 ± 10.5 years old, and 82.9% of the patients were male. The average values of the TyG index and SHR were 8.8 ± 0.8 and 0.9 ± 0.2, respectively. Compared with survivors, those who developed clinical events during the follow-up tended to be older, have hypertension, HF (lower LVEF), renal dysfunction (lower eGFR), prior MI, and prior stroke, and hold higher TyG index and SHR values. Regarding the lesions and treatment characteristics, patients with clinical events were more likely to have multivessel coronary disease, receive more stent treatment and have a lower percentage of procedural success. In addition, the baseline characteristics grouped by the TyG index and SHR levels are shown in Additional file 1: Tables S3, S4, respectively.

**Table 1:** Baseline patient characteristics grouped by outcomes

	All (n = 2740)	Survivors (n = 2571)	Patients with outcomes (n = 169)	*P*-value
Age, years	58.5 ± 10.5	58.3 ± 10.5	62.2 ± 10.6	< 0.001
Male, n (%)	2272 (82.9)	2139 (83.2)	133 (78.7)	0.132
BMI, kg/m^2^	26.3 ± 3.3	26.3 ± 3.3	25.8 ± 3.5	0.042
Smoking, n (%)	880 (32.1)	826 (32.1)	54 (32)	0.962
Prediabetes, n (%)	999 (36.5)	943 (36.7)	56 (33.1)	0.354
Diabetes, n (%)	1280 (46.7)	1193 (46.4)	87 (51.5)	0.200
Hypertension, n (%)	1777 (64.9)	1643 (63.9)	134 (79.3)	< 0.001
Dyslipidemia, n (%)	2182 (79.6)	2046 (79.6)	136 (80.5)	0.780
HF, n (%)	211 (7.7)	185 (7.2)	26 (15.4)	< 0.001
LVEF, (%)	59.9 ± 8.7	60.1 ± 8.5	57.3 ± 10.8	< 0.001
Prior MI, n (%)	901 (32.9)	836 (32.5)	65 (38.5)	0.111
Prior stroke, n (%)	350 (12.8)	320 (12.4)	30 (17.8)	0.045
Peripheral vascular disease, n (%)	188 (6.9)	173 (6.7)	15 (8.9)	0.285
Prior revascularization, n (%)	870 (31.8)	810 (31.5)	60 (35.5)	0.280
PCI	822 (30.0)	765 (29.8)	57 (33.7)	0.275
CABG	86 (3.1)	78 (3)	8 (4.7)	0.220
Renal dysfunction, n (%)	129 (4.7)	108 (4.2)	21 (12.4)	< 0.001
Unstable angina, n (%)	1162 (42.4)	1092 (42.5)	70 (41.4)	0.788
AMI, n (%)	233 (8.5)	220 (8.6)	13 (7.7)	0.696
Silent ischeamia, n (%)	541 (19.7)	501 (19.5)	40 (23.7)	0.186
Laboratory tests				
Hemoglobin, g/L	4.9 (4.5, 5.2)	4.9 (4.5, 5.2)	4.7 (4.4, 5.3)	0.269
Platelet, × 10^9^/L	0.4 (0.3, 0.5)	0.4 (0.3, 0.5)	0.4 (0.3, 0.5)	0.902
HbA1C, %	6.5 ± 1.2	6.5 ± 1.2	6.6 ± 1.1	0.669
FBG, mmol/L	6.6 ± 2.4	6.6 ± 2.4	7.5 ± 3.0	< 0.001
TC, mmol/L	3.8 (3.3, 4.6)	3.8 (3.3, 4.6)	3.9 (3.3, 4.6)	0.75
TG, mmol/L	1.4 (1.0, 2.0)	1.4 (1.0, 2.0)	1.5 (1.2, 2.0)	0.116
LDL-C, mmol/L	2.2 (1.8, 2.9)	2.2 (1.8, 2.9)	2.3 (1.8, 2.9)	0.784
HDL-C, mmol/L	1.0 (0.9, 1.2)	1.0 (0.9, 1.2)	1.0 (0.8, 1.2)	0.308
eGFR, mL/min/1.73 m^2^	98.8 ± 26.2	99.3 ± 25.9	91.3 ± 29.9	< 0.001
Hs-CRP, mmol/L	2.7 ± 3.2	2.7 ± 3.2	3.5 ± 3.6	0.005
TyG index	8.8 ± 0.8	8.8 ± 0.8	9.0 ± 0.7	< 0.001
SHR	0.9 ± 0.2	0.8 ± 0.2	0.9 ± 0.3	< 0.001
Angiographic characteristics, n (%)				
Multivessel disease	2395 (87.4)	2238 (87)	157 (92.9)	0.026
Ostial lesion	440 (16.1)	413 (16.1)	27 (16)	0.976
Bifurcation	944 (34.5)	893 (34.7)	51 (30.2)	0.227
Intervention treatment, n (%)				
LM	37 (1.4)	37 (1.4)	0 (0.0)	0.167
LAD	940 (34.3)	889 (34.6)	51 (30.2)	0.243
LCX	377 (13.8)	358 (13.9)	19 (11.2)	0.327
RCA	1367 (49.9)	1273 (49.5)	94 (55.6)	0.124
Graft	7 (0.3)	6 (0.2)	1 (0.6)	0.360
Number of lesions ≥ 2	732 (26.7)	689 (26.8)	43 (25.4)	0.700
Number of stents ≥ 2	1546 (56.4)	1470 (57.2)	76 (45)	0.002
Procedural success	2233 (81.5)	2105 (81.9)	128 (75.7)	0.047
Medication at discharge, n (%)				
DAPT	2716 (99.1)	2548 (99.1)	168 (99.4)	1.000
Aspirin	2740 (100.0)	2571 (100)	169 (100)	1.000
Clopidogrel	2539 (92.7)	2379 (92.5)	160 (94.7)	0.301
Ticagrelor	599 (21.9)	570 (22.2)	29 (17.2)	0.127
β-blockers	2509 (91.6)	2358 (91.7)	151 (89.3)	0.284
Statin	2659 (97.0)	2495 (97.0)	164 (97.0)	1.000
Antidiabetic agents	970 (35.4)	905 (35.2)	65 (38.5)	0.390

*Tyg* triglyceride-glucose, *CV* cardiovascular, *TVMI* target vessel myocardial infarction, *MACCEs*, major adverse CV cerebral events, *SHR* stress-hyperglycemia ratio, *BMI* body mass index, *T2DM* type 2 diabetes mellitus, *HF* heart failure, *LVEF* left ventricular ejection faction, *AMI* acute myocardial infarction, *PCI* percutaneous coronary intervention, *CABG* coronary artery bypass grafting, *PAD* peripheral artery disease, *TC* total cholesterol, *HDL-C* high-density lipoprotein cholesterol, *LDL-C* low-density lipoprotein cholesterol, *HbA1c* glycosylated hemoglobin *A1c* *FBG* fasting blood glucose, *hs* *CRP* high-sensitivity C-reactive protein, *eGFR* estimated glomerular filtration rate, *LM* left main, *DAPT* dual antiplatelet therapy

### The TyG index and the long-term prognosis in CTO patients

**Fig. 2:**
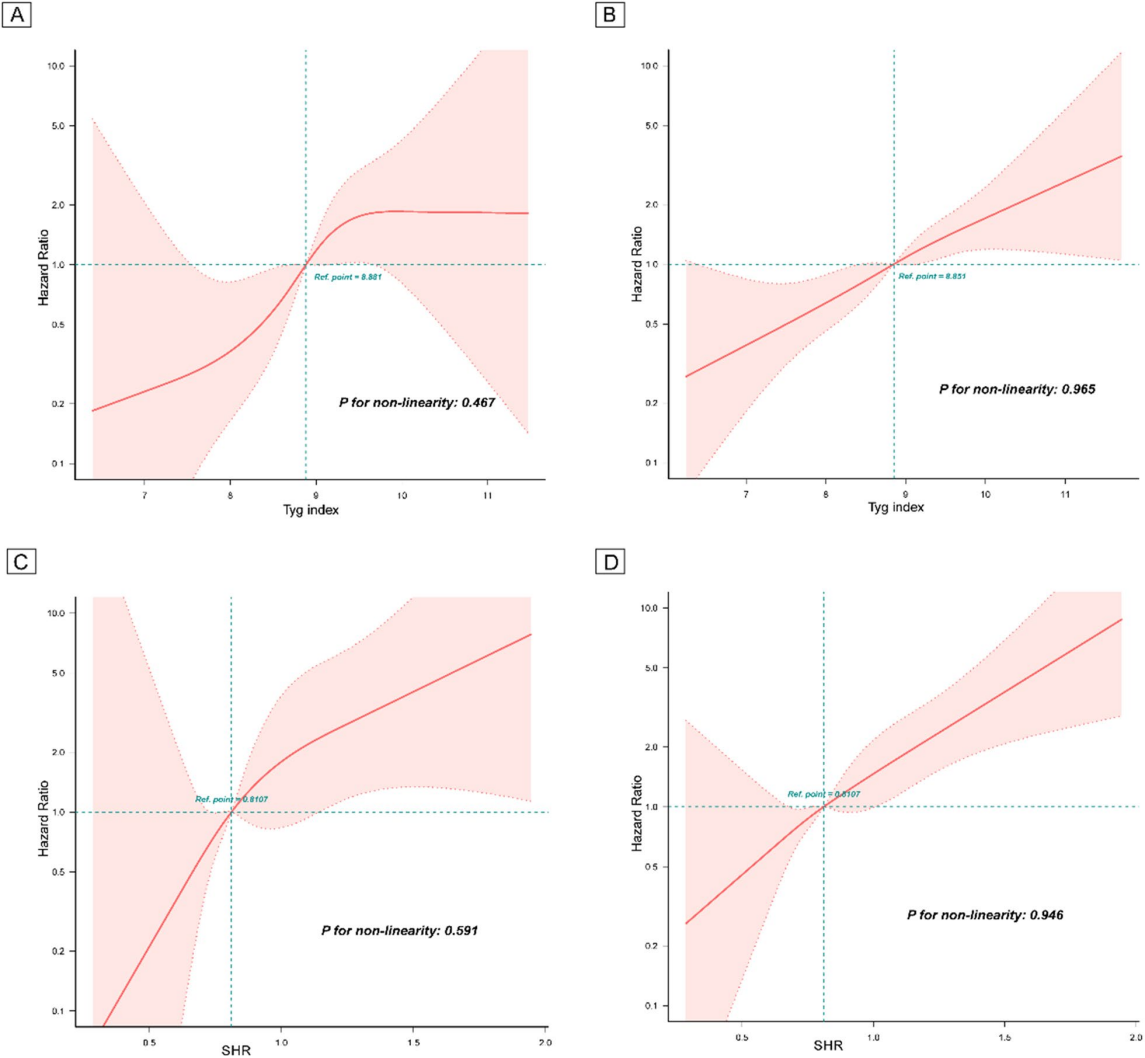
RCS curves for the association of TyG index with the risk of CV death and TVMI **A**, the risk of MACCEs B, the association of SHR with the risk of CV death and TVMI **C**, and the risk of MACCEs **D** *RCS* Restricted cubic spline, *TyG* triglyceride-glucose, *CV* cardiovascular, *TVMI* target vessel myocardial infarction, *MACCEs* major adverse CV cerebral events, *SHR* stress-hyperglycemia ratio

**Fig. 3:**
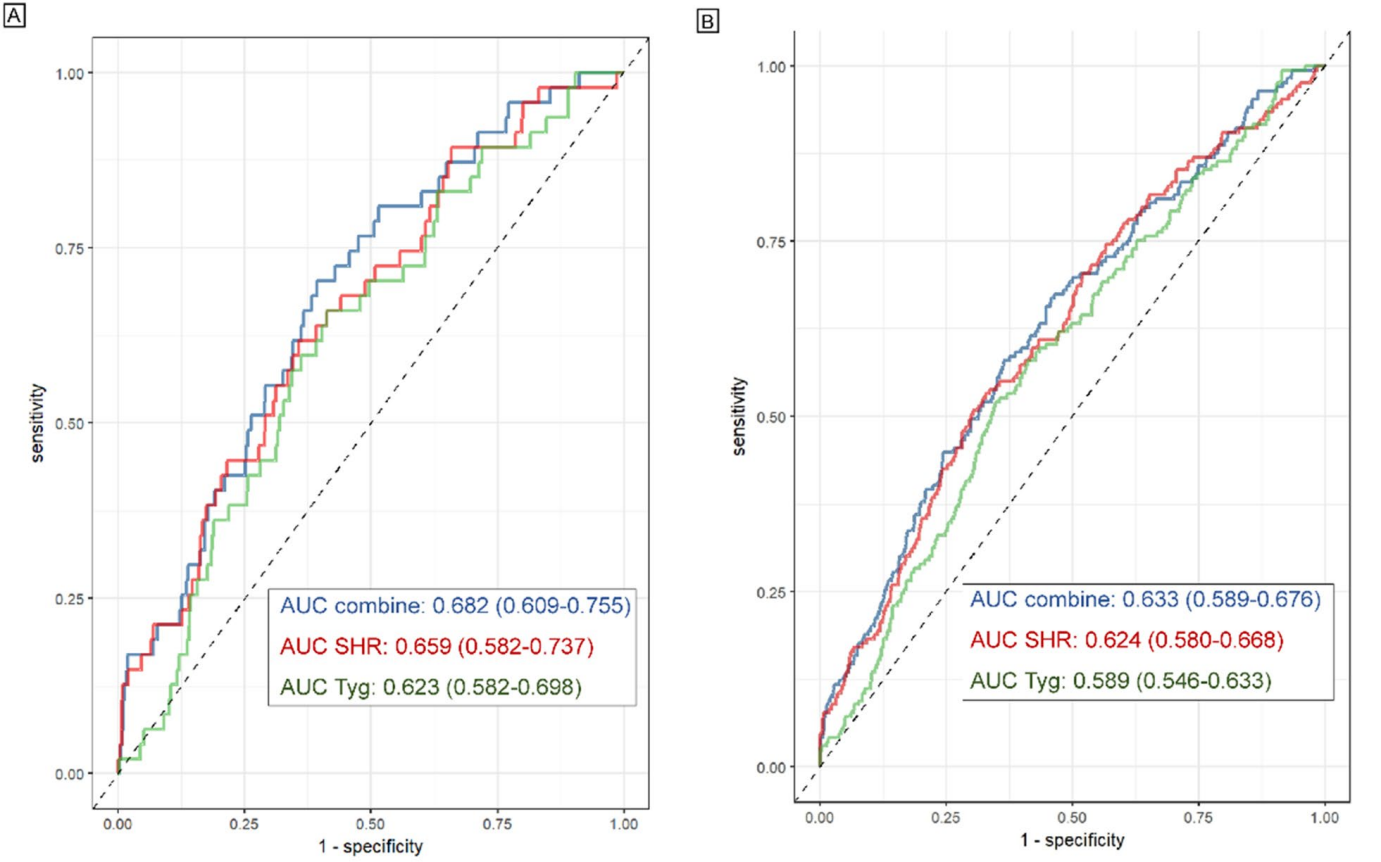
ROC curves for the use of TyG index, SHR, and their combination in predicting the prognosis of CTO patients **A** for the composite of CV death and TVMI; Fig. 3B: for MACCEs. Optimal cut-off: (1) the composite of CV death and TVMI: TyG, 8.98; SHR: 0.86; (2) MACCE: TyG, 9.07; SHR: 0.88 *ROC *receiver operating characteristic, *AUC* an area under the curve, *TyG* triglyceride-glucose, *CV* cardiovascular, *TVMI* target vessel myocardial infarction, *MACCEs* major adverse CV cerebral events, *SHR* stress-hyperglycemia ratio

During the follow-up with a median time of 3 years, 179 (6.5%) cases of MACCEs, including 47 (1.7%) cases of CV death or TVMI, were recorded. The association of the TyG index with CV risk is shown in Table 2. Compared with patients with a low TyG index, those with a high TyG index showed a significantly higher risk of both CV death/TVMI (T3 vs. T1: HR 4.23, 95% CI 1.58–11.37) and MACCEs (T3 vs. T1: HR 2.47, 95% CI 1.54–3.97). KM plots for groups with different TyG index values revealed that the group with the highest TyG index values showed the highest risk of both CV death/TVMI and MACCEs (log-rank *P* < 0.001) **(**Additional file 1: Figure S1A, D). When analysed, the TyG index is a continuous variable, with the increase in per unit in the TyG index, the risk of CV death/TVMI and MACCEs elevated by 97% and 73%, respectively **(**Table 2**)**. RCS curves showed that the TyG index was positively linearly correlated with the risk of CV death/TVMI and MACCEs (*P* for nonlinearity = 0.467 and 0.965) **(**Fig. 2A, B**)**. The survival analysis also showed that a high TyG index was significantly associated with an increased risk of all-cause death (T3 vs. T1: HR 2.22, 95% CI 1.24–3.95) (Additional file 1: Table S5). For the predictive value of the TyG index for CV death/TVMI and MACCEs, the ROC curves showed that the AUCs of CV death/TVMI and MACCEs evaluated by the TyG index were 0.623 (95% CI: 0.582–0.698) and 0.589 (95% CI: 0.546–0.633), respectively **(**Table 3**, **Fig. 3**)**. The optimal cut-off value of the TyG index was 8.98 for CV death/TVMI and 9.07 for MACCEs **(**Table 4**, **Fig. 3**)**. The correlation between the TyG index and TC, LDL-C, and hs-CRP is presented in Additional file 1: Table S6, which shows that the TyG index was significantly positively correlated with TC (R^2^ 0.64, 95% CI 0.56–0.73) and LDL-C levels (R^2^ 0.25, 95% CI 0.18–0.33).

**Table 2:** Cox regression models for the association of the TyG index and SHR with clinical outcomes

	Per one unit increase^a^	Groups			*P* for trend
		T1	T2	T3	
TyG index					
CV death and TVMI					
No/Subject		7/895	13/917	27/928	
Crude	1.67 (1.15–2.43)	1.00	1.78 (0.71–4.47)	3.74 (1.62–8.56)	0.001
Model 1	1.94 (1.30–2.90)	1.00	2.05 (0.81–5.16)	4.76 (2.04–11.11)	< 0.001
Model 2	2.15 (1.36–3.40)	1.00	1.90 (0.75–4.83)	5.07 (2.10–12.23)	< 0.001
Model 3	1.97 (1.19–3.27)	1.00	1.48 (0.53–4.09)	4.23 (1.58–11.37)	0.004
MACCEs					
No/Subject		40/895	56/917	83/928	
Crude	1.50 (1.23–1.82)	1.00	1.10 (0.72–1.68)	2.04 (1.40–2.98)	< 0.001
Model 1	1.62 (1.32–2.00)	1.00	1.19 (0.77–1.82)	2.35 (1.60–3.45)	< 0.001
Model 2	1.67 (1.34–2.08)	1.00	1.17 (0.76–1.79)	2.46 (1.64–3.68)	< 0.001
Model 3	1.73 (1.34–2.24)	1.00	1.31 (0.81–2.14)	2.47 (1.54–3.97)	< 0.001
SHR					
CV death and TVMI					
No/Subject		5/897	15/911	27/932	
Crude	1.28 (1.15–1.41)	1.00	2.94 (1.07–8.08)	5.18 (1.99–13.45)	< 0.001
Model 1	1.25 (1.14–1.38)	1.00	3.09 (1.12–8.51)	5.54 (2.13–14.40)	< 0.001
Model 2	1.29 (1.15–1.44)	1.00	2.88 (1.04–8.01)	5.70 (2.16–15.04)	< 0.001
Model 3	1.22 (1.14–1.32)	1.00	2.53 (0.87–7.39)	5.14 (1.89–13.98)	0.001
MACCEs					
No/Subject		31/897	48/911	90/932	
Crude	1.23 (1.16–1.30)	1.00	1.51 (0.96–2.37)	2.87 (1.91–4.32)	< 0.001
Model 1	1.22 (1.15–1.29)	1.00	1.56 (0.99–2.45)	2.98 (1.98–4.48)	< 0.001
Model 2	1.23 (1.16–1.32)	1.00	1.55 (0.98–2.44)	3.04 (2.01–4.61)	< 0.001
Model 3	1.26 (1.11–1.44)	1.00	1.58 (0.96–2.59)	2.91 (1.84–4.60)	< 0.001

Model 1: adjusted for age, sex, and BMI

Model 2: adjusted for age, sex, BMI, smoking, T2DM, dyslipidemia, hypertension, prior HF, prior MI, prior stroke, peripheral vascular disease, prior revascularization, and AMI

Model 3: adjusted for age, sex, BMI, smoking, HF, T2DM, dyslipidemia, hypertension, prior MI, prior stroke, peripheral vascular disease, prior revascularization, AMI, multivessel disease, ostial lesion, bifurcation, number of lesions ≥ 2, number of stents ≥ 2, procedural success, eGFR, DAPT, statin, and anti-diabetic drug

*TyG* triglyceride-glucose, *CV* cardiovascular, *eGFR* estimated glomerular filtration rate, *MI* myocardial infarction, *MACCEs* major adverse cardiovascular cerebral events, *SHR* stress-hyperglycemia ratio, *BMI* body mass index, *T2DM* type 2 diabetes mellitus, *HF* heart failure, *AMI* acute myocardial infarction, *DAPT* dual anti-platelet therapy

^a^Per one unit refers to “1.00” in the Tyg index and “0.10” in SHR

**Table 3:** Cox regression models for the association of the combination of Tyg index and SHR with clinical outcomes

	Groups				*P* for trend
	Low TyG and low SHR	Low TyG and high SHR	High TyG and low SHR	High TyG and high SHR	
CV death and TVMI					
No/subject	13/1353	7/460	7/455	20/472	
Crude	1.00	1.55 (0.62–3.89)	1.60 (0.64–4.00)	4.49 (2.23–9.02)	< 0.001
Model 1	1.00	1.67 (0.67–4.19)	2.01 (0.79–5.08)	5.09 (2.51–10.29)	< 0.001
Model 2	1.00	1.96 (0.77–5.00)	2.35 (0.91–6.08)	6.40 (2.92–14.05)	< 0.001
Model 3	1.00	2.24 (0.82–6.13)	2.42 (0.83–7.06)	7.16 (2.90–17.69)	< 0.001
MACCEs					
No/subject	60/1353	26/460	19/455	64/472	
Crude	1.00	1.26 (0.79–1.99)	1.02 (0.55–1.55)	3.28 (2.30–4.66)	< 0.001
Model 1	1.00	1.29 (0.81–2.05)	1.05 (0.62–1.76)	3.54 (2.48–5.06)	< 0.001
Model 2	1.00	1.38 (0.87–2.2)	1.16 (0.68–1.96)	4.07 (2.75–6.02)	< 0.001
Model 3	1.00	1.48 (0.88–2.47)	1.25 (0.71–2.19)	3.62 (2.32–5.64)	< 0.001

Model 1: adjusted for age, sex, and BMI

Model 2: adjusted for age, sex, BMI, smoking, T2DM, dyslipidemia, hypertension, prior HF, prior MI, prior stroke, peripheral vascular disease, prior revascularization, and AMI

Model 3: age, sex, BMI, smoking, HF, T2DM, dyslipidemia, hypertension, prior MI, prior stroke, peripheral vascular disease, prior revascularization, AMI, multivessel disease, ostial lesion, bifurcation, number of lesions ≥ 2, number of stents ≥ 2, procedural success, eGFR, DAPT, statin, and anti-diabetic drug

Low TyG index or SHR: T1-2; High Tyg index or SHR: T3

*TyG* triglyceride-glucose, *CV* cardiovascular, *eGFR* estimated glomerular filtration rate, *MI *myocardial infarction, *MACCEs* major adverse cardiovascular cerebral events, *SHR* stress-hyperglycemia ratio*, BMI* body mass index, *T2DM* type 2 diabetes mellitus, *HF* heart failure, *AMI *acute myocardial infarction, *DAPT* dual anti-platelet therapy

### SHR and the long-term prognosis in CTO patients

As shown in Table 2, CTO patients with high SHR levels presented a significantly higher risk of CV death/TVMI (T3 vs. T1: HR 5.14, 95% CI 1.89–13.98) and MACCEs (T3 vs. T1: HR 2.91, 95% CI 1.84–4.60) than those with low SHR levels. KM plots also confirmed the highest risk of clinical outcomes in CTO patients with the highest SHR tertile (log-rank *P* < 0.001) (Additional file 1: Figure S1B, E). In addition, the risks of CV death/TVMI and MACCEs were shown to increase by 22% and 26%, respectively, per 0.1 unit increase in SHR **(**Table 2**)**. RCS curves also showed a positive linear correlation between SHR and CV death/TVMI and MACCEs (*P* for nonlinearity = 0.591 and 0.946) **(**Fig. 2C, D**)**. Compared with those with a low SHR, patients with a high SHR also showed an increased risk of all-cause death in the survival analysis (T3 vs. T1: HR 2.79, 95% CI 1.44–5.42) (Additional file 1: Table S5). The AUCs of CV death/TVMI and MACCEs evaluated by the SHR were 0.659 (95% CI 0.582–0.737) and 0.624 (95% CI 0.580–0.668), respectively, and the optimal cut-off value of the SHR was 0.86 for CV death/TVMI and 0.88 for MACCEs (Table 4, Fig. 3). Additional file 1: Table S6 shows that the SHR was positively correlated with TC (R^2^ 0.30, 95% CI 0.04–0.56) and hs-CRP levels (R^2^ 0.94, 95% CI 0.05–1.28) with statistical significance.

### The association of the TyG index and SHR with long-term prognosis in CTO patients under different glucose metabolic statuses and procedural outcomes

When analysing patients with different glucose metabolic statuses (NG, prediabetes, and T2DM), we found that the positive association between a high TyG index and elevated long-term risk of clinical outcomes was particularly significant in patients with T2DM (Additional file 1: Table S7). For the SHR, it was shown that the association of a high SHR with increased CV risk was especially significant in patients with prediabetes and T2DM (Additional file 1: Table S7). We further analysed patients with different procedural outcomes (procedural success and failure) and determined that the positive association of both the TyG index and SHR with long-term CV risk was significant regardless of procedural outcomes (Additional file 1: Table S8).

### The combination of the TyG index and SHR and the long-term prognosis in CTO patients

In Table 3**,** we show that patients with both a high TyG index and high SHR (Tertile 3) showed a significantly higher risk of CV death/TVMI (HR 7.16, 95% CI 2.90–17.69) and MACCEs (HR 3.62, 95% CI 2.32–5.64) when compared with those with low levels (Tertiles 1–2). KM plots showed that the group with both a high TyG index and SHR levels (Tertile 3) presented the highest risk of clinical outcomes during the follow-up **(**Additional file 1: Figure S1C, F). ROC curves of the TyG index-SHR combination showed that its AUCs of CV death/TVMI and MACCEs were 0.682 (95% CI 0.609–0.755) and 0.633 (95% CI 0.589–0.676), respectively (Fig. 3). Comparisons of predictive values of the TyG index, SHR, and TyG index-SHR combination for CV death/TVMI and MACCEs are shown in Table 4. The results showed that the predictive value of the TyG index-SHR combination was higher than that of the TyG index and SHR itself, although statistical significance was only present in the comparison between the TyG index-SHR combination and the TyG index for the risk of MACCEs (△AUC = 0.043, *P* value = 0.014) (Table 4). Survival analysis showed that patients with elevated levels of both TyG index and SHR exhibited the highest risk of mortality compared to the other groups with varying levels of TyG and SHR (Additional file 1: Table S9).

**Table 4:** Comparisons among the predictive value of the TyG index, SHR, and combined model for clinical outcomes

	AUC/△AUC	95% CI	*P* value
CV death and TVMI			
AUC combined	0.682	0.609–0.755	–
AUC SHR	0.659	0.582–0.737	–
AUC TyG	0.623	0.582–0.698	–
AUC combined–AUC TyG	0.058	− 0.006–0.121	0.074
AUC combined–AUC SHR	0.022	− 0.015–0.060	0.247
AUC SHR– AUC TyG	0.036	− 0.057–0.128	0.452
MACCEs			
AUC combined	0.633	0.589–0.676	–
AUC SHR	0.624	0.580–0.668	–
AUC TyG	0.589	0.546–0.633	–
AUC combined–AUC TyG	0.043	0.009–0.077	0.014
AUC combined–AUC SHR	0.009	− 0.013–0.030	0.426
AUC SHR– AUC TyG	0.034	− 0.084–0.016	0.179

Optimal cut-off: (1) the composite of CV death and TVMI: TyG, 8.98; SHR: 0.86; (2) MACCE: TyG, 9.07; SHR: 0.88

*TyG* triglyceride-glucose, *CV* cardiovascular, *TVMI* target vessel myocardial infarction, *MACCEs* major adverse CV cerebral events, *SHR* stress-hyperglycemia ratio

### Subgroup and sensitivity analyses

Subgroup analyses of the association of the TyG index and SHR with clinical outcomes were performed according to age (≥ 60 years old), sex, BMI (≥ 30 kg/m^2^), smoking status, hyperlipidaemia, hypertension, HF with low LVEF (< 50%), renal dysfunction, and AMI, which showed consistent trends with the main results and no significant interaction in all subgroups (Additional file 1: Table S10–S13). In the sensitivity analysis, we excluded patients who presented clinical events within 90 days, and the association of the TyG index and SHR with clinical outcomes was still significant (Additional file 1: Table S14).

## Discussion

The current large-population-based prospective cohort study included 2740 patients with CTO, conducted a clinical follow-up with a median time of 3 years, and investigated the association of the TyG index and SHR with the long-term prognosis. The major findings of this investigation included the following: 1) high Tyg index and SHR levels were related to an increase in the long-term risk of adverse events in CTO patients; 2) the association of the TyG index with high CV risk was especially significant in patients with T2DM, while the association of SHR with high CV risk was significant in patients with both prediabetes and T2DM; 3) the association of the TyG index and SHR with high CV risk was significant regardless of the procedural outcomes; and 4) patients with both high TyG index and SHR showed the worst prognosis among people with different levels of these two biomarkers, and the predictive value of the TyG index-SHR combination for the risk of MACCEs was more significant than the TyG index itself.

The TyG index has been widely demonstrated as a reliable marker to assess insulin resistance with high sensitivity and specificity, and it has been widely applied in clinical practice for its convenience, low cost, and wide-range usage [19]. The association between Tyg index levels and CVD was comprehensively discussed, and its predictive value for a high incidence of CHD has been extensively revealed in previous cohort studies and meta-analyses [20, 21]. Regarding the prognostic value of the TyG index in CHD patients, several cohort studies showed that patients with a high TyG index were independently associated with a high risk of repeat revascularization and hospital mortality [22, 23]. With respect to CTO, the prognostic role of the TyG index in patients with CTO lesions was previously investigated by several studies; however, the sample size of these studies was not satisfactory, and the evidence was not sufficient. In 2022, Li et al. conducted a rigorous retrospective cohort study and reported that a high TyG index was significantly related to an elevated CV risk [9]. Although that was a well-conducted study, there were only 652 CTO patients enrolled [9]. That small sample size might have potentially biased the results. Compared with the study conducted by Li et al. we not only validated the positive association of a high TyG index with poor prognosis in a much larger population (2740 patients) but also revealed the AUC and optimal cut-off of the TyG index in predicting CV adverse events in the current study. In addition, we conducted a subgroup analysis for this association in patients with differing glycaemic status and showed that the association of a high TyG index with poor prognosis was only significant among patients with T2DM, which was consistent with previous studies [9, 24]. Notably, we found that some results of this study differed from those of Li’s study. First, our subgroup analysis showed that the TyG index was significantly correlated with CV risk in patients with both successful and failed PCI procedures, while this correlation was only reported among patients who received successful CTO PCI in Li’s study [9]. Moreover, the RCS curves for the relationship between the TyG index and the risk of CV adverse events in our study showed a positive linear type, while Li et al. reported a “J”-shape relationship for the association between the TyG index and CV risk [9]. This kind of difference might be attributed to the discrepancy in sample size, and it is difficult to compare the accuracy of the conclusions from both studies due to the insufficiency of related research [9]. Therefore, further studies are needed to explore the association between the TyG index and CV risk in CTO patients in large, randomized controlled trials (RCTs).

Another focus of the current study is the association of the SHR with long-term prognosis in patients with CTO lesions. Ample literature suggests that the SHR could better reflect blood glucose status than FBG or HbA1C [5, 25]. Recently, scholars identified the SHR as an independent predictor of high CV risk in patients with CVD, especially for those with acute diseases such as acute coronary disease [26], AMI [27], critical HF [28], and MI with nonobstructive coronary arteries [29]. For chronic diseases, Xu et al. focused on patients with chronic coronary syndrome and reported a significant association of a high SHR with elevated in-hospital mortality, further indicating the potential prognostic value of the SHR in patients without stress conditions [30]. For patients with CTO lesions, there is no research elucidating the association of the SHR with long-term prognosis thus far. Herein, we focused on this point for the first time and revealed that a high SHR was associated with an increase in long-term CV risk in CTO patients undergoing PCI. Interestingly, the association with the SHR seems to be different in CHD patients with differing glycaemic status (NG, prediabetes, and T2DM). Zhang et al. conducted a retrospective cohort with 987 CHD patients and reported that a high SHR correlated with the improved risk of multivessel CHD in the prediabetes and DM groups, but this correlation was not significant in patients with NG [8]. Similar to Zhang’s study, we also determined that the predictive value of the SHR for poor prognosis in patients with CTO lesions was especially significant in those with prediabetes and T2DM. However, we reported different conclusions in a previous investigation, as we found that the SHR was significantly correlated with an increase in 2-year mortality in both diabetic and nondiabetic patients with AMI [31]. This paradox is potentially attributed to the different target diseases in the two studies (AMI and CTO). We speculated that the SHR in a patient with AMI might be mostly indicative of acute disease-related stress, and the stress extent, which was not affected greatly by basal glucose metabolism, had effects on patients with and without diabetes on an equal basis. The SHR in CTO patients was speculated to mostly reflect true glucose metabolism disorders and inflammation burden (as we showed that SHR was significantly correlated with levels of hs-CRP), which were more severe in patients with prediabetes and T2DM. Therefore, the relationship between the SHR and glycaemic status might be different in patients with different diseases. Nevertheless, this hypothesis has not yet been confirmed, and we anticipate that further studies could report more rigorous evidence.

This is the first study to combine the TyG index and the SHR and explore their combined predictive value for poor prognosis in patients with CHD. As the results showed in our study, patients with both a high TyG index and a high SHR presented a high risk of both CV death/TVMI and MACCEs, and the ROC curves showed that the AUC of the TyG index-SHR combination for predicting MACCE risk was larger than that of the TyG index itself. We speculated that this phenomenon was attributed to complementary effects between the TyG index and the SHR, as the TyG index mainly evaluated the extent of insulin resistance and the SHR could additionally reflect inflammation burden and glucose metabolism [32, 33]. However, since we only included patients with CTO lesions and no previous research combined the TyG index and the SHR, the predictive advantage of the TyG index-SHR combination cannot be confirmed in patients with CHD or other CVD diseases. This conclusion is expected to be validated among different populations.

HF and renal disease are recognized as significant factors contributing to the poor prognosis of patients with CTO. Previous studies have demonstrated that CTO patients with HF experience higher in-hospital mortality rates and a greater number of complications [34]. Similarly, renal disease, particularly contrast nephropathy, has been associated with increased mortality in CTO patients [35–37]. Consequently, identifying biomarkers with strong prognostic value in CTO patients with HF or renal disease is of great importance. In this study, we determined that a high TyG index and a high SHR were associated with an increased risk of adverse cardiac events in CTO patients with HF or renal disease, further strengthening the prognostic values of these two biomarkers. It is important to note that the specific type of nephropathy was not recorded in our study, thus limiting our ability to determine the extent to which the prognostic value of these biomarkers applies to CTO patients with contrast nephropathy. Future investigations should aim to address this knowledge gap.

There are several limitations to this study. First, the single-centre enrolment and the Chinese-only population limited the representativeness of this investigation. Second, the dynamic change in the TyG index and the SHR during follow-up was not presented in our study; thus, we are not able to evaluate the impact of changes in these two biomarkers on the prognosis. Third, clinical follow-up for angiographic results was not conducted in this study, which lacked the specific impact of the TyG index and the SHR on coronary lesions. Fourth, although we have adjusted potential confounders as covariates in multivariable regression models, the influence of uncollected confounders cannot be dismissed entirely. Fifth, it is worth noting that our study did not include a follow-up evaluation of the quality of life. Considering that one of the primary objectives of PCI in patients with CTO is to alleviate ischaemic symptoms, such as angina, it would be valuable to investigate the impact of a high TyG index and SHR on the quality of life in future studies. Finally, no randomization was included in our study; therefore, the conclusions of this study warrant validation in future RCTs.

## Conclusion

This study, for the first time, confirmed the predictive value of the TyG index and the SHR for poor prognosis in patients with CTO lesions in a large population and verified conclusions in patients with differing glycaemic status and procedural outcomes. In addition, we combined the TyG index and the SHR and found that their combination had a more significant predictive value for CV risk. These findings indicated that the TyG index and the SHR held great prognostic value for patients with CTO lesions and thus supported the use of these two biomarkers as potential tools in identifying patients with high risk and guiding further treatment or follow-up strategies.

## Supplementary Information

**Additional file 1: Table S1.** CV death and target vessel Mi risk according to baseline variables. **Table S2.** MACCE risk according to baseline variables. **Table S3.** Baseline patient characteristics grouped by TyG index levels. Table S4. Baseline patient characteristics grouped by SHR levels. **Table S5.** Survival analysis for the association of the TyG index and SHR with the risk of all-cause death. **Table S6.** Correlation analysis between the TyG index/SHR and TC, LDL-C, and hs-CRP. **Table S7.** Cox regression models for the association of TyG index and SHR with clinical outcomes in patients with different glucose statuses. **Table S8.** Cox regression models for the association of TyG index and SHR with clinical outcomes in patients with different procedural outcomes. **Table S9.** Survival analysis for the association of the combination of Tyg index and SHR with the risk of all-cause death. **Table S10.** Subgroup analyses of the associations between TyG index and the risk of the composite of CV death and target vessel MI. **Table S11.** Subgroup analyses of the associations between TyG index and the risk of MACCEs. **Table S12.** Subgroup analyses of the associations between SHR and the risk of the composite of CV death and target vessel MI. **Table S13.** Subgroup analyses of the associations between SHR and the risk of MACCEs. **Table S14.** Sensitivity analyses of the associations of TyG index and SHR with outcomes after excluding patients who presented clinical events within 90 days (n=2724). **Figure S1.** Kaplan–Meier curves for the cumulative incidence of clinical outcomes in patients grouped by the TyG index (Figure S1A: CV death and TVMI; Figure S1D: MACCEs), SHR (Figure S1B: CV death and TVMI; Figure S1E: MACCEs), and combination of two ratios (Figure S1C: CV death and TVMI; Figure S1F: MACCEs).

## Data Availability

The datasets used during the current study are available from the corresponding author on reasonable request.

## Abbreviations

AMI: Acute myocardial infarction
AUC: Reas under the curve
BMI: Body mass index
CV: Cardiovascular
CTO: Chronic total occlusion
CIs: Confidence intervals
CABG: Coronary artery bypass grafting
DAPT: Dual antiplatelet therapy
eGFR: Estimated glomerular filtration rate
FBG: Fasting blood glucose
HF: Heart failure
HDL-C: High-density lipoprotein cholesterol
HbA1c: Glycosylated hemoglobin A1c
HRs: Hazard ratios
hs CRP: High-sensitivity C-reactive protein
LVEF: Left ventricular ejection faction
LDL-C: Low-density lipoprotein cholesterol
LM: Left main
MACCEs: Major cardiovascular cerebrovascular adverse events
PCI: Percutaneous coronary intervention
PAD: Peripheral artery disease
RCSs: Restricted cubic splines
ROC: Receiver operating characteristic
SHR: Stress hyperglycaemia ratio
TyG: Triglyceride-glucose
TVMI: Target vessel myocardial infarction
T2DM: Type 2 diabetes mellitus
TC: Total cholesterol
