# Editorial Expression of Concern: High triglyceride‑glucose index and stress hyperglycemia ratio as predictors of adverse cardiac events in patients with coronary chronic total occlusion: a large‑scale prospective cohort study

**DOI:** 10.1186/s12933-025-03045-4

**Published:** 2025-12-23

**Authors:** Yanjun Song, Kongyong Cui, Min Yang, Chenxi Song, Dong Yin, Qiuting Dong, Ying Gao, Kefei Dou

**Affiliations:** 1 Cardiometabolic Medicine Center, Department of Cardiology, National Center for Cardiovascular Diseases, Fuwai Hospital, Chinese Academy of Medical Sciences and Peking Union Medical College, Beijing, China; 2 State Key Laboratory of Cardiovascular Disease, 167, Beilishi Road, Xicheng District, Beijing, 100037 China

**Editorial Expression of Concern to: Cardiovascular Diabetology (2023) 22:180** 10.1186/s12933-023-01883-8

The Editor-in-Chief is issuing this editorial expression of concern to alert readers that the authors mistakenly listed calculation of the TyG index in mmol/l rather than the correct mg/dl.

Yanjun Song, Kongyong Cui, Min Yang, Qiuting Dong, Ying Gao and Kefei Dou agree to this Editorial Express of Concern. Chenxi Song and Dong Yin did not respond to correspondence from the Publisher about this Editorial Express of Concern.

