# Supplementary material for: High triglyceride-glucose index and stress hyperglycemia ratio as predictors of adverse cardiac events in patients with coronary chronic total occlusion: a large-scale prospective cohort study
Source: Cardiovasc Diabetol. 2023 Jul 15;22:180. doi: 10.1186/s12933-023-01883-8 (PMC10350280; doi:10.1186/s12933-023-01883-8)
Supplement: Supplementary file 1 — Additional file 1: Table S1. CV death and target vessel Mi risk according to baseline variables. Table S2. MACCE risk according to baseline variables. Table S3. Baseline patient characteristics grouped by TyG index levels. Table S4. Baseline patient characteristics grouped by SHR levels. Table S5. Survival analysis for the association of the TyG index and SHR with the risk of all-cause death. Table S6. Correlation analysis between the TyG index/SHR and TC, LDL-C, and hs-CRP. Table S7. Cox regression models for the association of TyG index and SHR with clinical outcomes in patients with different glucose statuses. Table S8. Cox regression models for the association of TyG index and SHR with clinical outcomes in patients with different procedural outcomes. Table S9. Survival analysis for the association of the combination of Tyg index and SHR with the risk of all-cause death. Table S10. Subgroup analyses of the associations between TyG index and the risk of the composite of CV death and target vessel MI. Table S11. Subgroup analyses of the associations between TyG index and the risk of MACCEs. Table S12. Subgroup analyses of the associations between SHR and the risk of the composite of CV death and target vessel MI. Table S13. Subgroup analyses of the associations between SHR and the risk of MACCEs. Table S14. Sensitivity analyses of the associations of TyG index and SHR with outcomes after excluding patients who presented clinical events within 90 days (n=2724). Figure S1. Kaplan–Meier curves for the cumulative incidence of clinical outcomes in patients grouped by the TyG index (Figure S1A: CV death and TVMI; Figure S1D: MACCEs), SHR (Figure S1B: CV death and TVMI; Figure S1E: MACCEs), and combination of two ratios (Figure S1C: CV death and TVMI; Figure S1F: MACCEs). [file 12933_2023_1883_MOESM1_ESM.docx]

**Supplementary Materials**

**High Triglyceride-Glucose Index and Stress Hyperglycemia Ratio as Predictors of Adverse Cardiac Events in Patients with Coronary Chronic Total Occlusion: A Large-Scale Prospective Cohort Study**

Yanjun Song^1,2†^, Kongyong Cui^1,2†^, Yang Min^1,2†^, Chenxi Song^1,2^, Dong Yin^1,2^, Qiuting Dong^1,2^, Ying Gao^1,2*^, Kefei Dou^1,2*^

^1^ State Key Laboratory of Cardiovascular Disease, Beijing, China.

^2^ Department of cardiology, Fuwai Hospital, National Center for Cardiovascular Diseases, Chinese Academy of Medical Sciences and Peking Union Medical College, 167A Beilishi Road, Xi Cheng District, Beijing 100037, China.

^†^Yanjun Song and Kongyong Cui contributed equally to this article.

^*^Kefei Dou and Ying Gao contributed equally as senior authors.

**Table S1. CV death and target vessel Mi risk according to baseline variables**

| Variables | HR (95%CI) | *P*-value |
| --- | --- | --- |
| Age (per one-year increase) | 1.1 (1.03-1.16) | 0.003 |
| Male (female as reference) | 0.94 (0.22-4.02) | 0.933 |
| BMI (per 1 kg/m^2^ increase) | 0.99 (0.85-1.17) | 0.949 |
| Smoking (no as reference) | 0.56 (0.16-2.03) | 0.379 |
| Prediabetes (no as reference) | 0.65 (0.15-2.85) | 0.566 |
| Diabetes (no as reference) | 1.02 (0.19-5.45) | 0.979 |
| Hypertension (no as reference) | 2.78 (0.71-10.93) | 0.143 |
| Dyslipidemia (no as reference) | 0.66 (0.2-2.19) | 0.498 |
| HF (no as reference) | 2.44 (1.01-5.94) | 0.048 |
| Prior MI (no as reference) | 0.54 (0.18-1.56) | 0.252 |
| Prior stroke (no as reference) | 0.91 (0.27-3.1) | 0.886 |
| PAD (no as reference) | 3.45 (1.1-10.85) | 0.034 |
| Renal dysfunction (no as reference) | 4.50 (1.50-13.53) | 0.007 |
| Prior revascularization (no as reference) | 0.16 (0.01-4.02) | 0.266 |
| PCI (no as reference) | 11.38 (0.52-249.62) | 0.123 |
| CABG (no as reference) | 11.94 (1.53-93.21) | 0.018 |
| Unstable angina (no as reference) | - (0-Inf) | 0.997 |
| AMI (no as reference) | 1.21 (0.2-7.09) | 0.837 |
| Silent ischeamia (no as reference) | - (0-Inf) | 0.998 |
| Hemoglobin (per 1 unit increase) | 1.15 (0.41-3.19) | 0.791 |
| Platelet (per 1 unit increase) | 1.01 (1-1.01) | 0.153 |
| HbA1C (per 1 unit increase) | 1.31 (0.61-2.81) | 0.492 |
| FBG (per 1 unit increase) | 0.85 (0.6-1.21) | 0.365 |
| TC (per 1 unit increase) | 0.05 (0.01-0.44) | 0.007 |
| TG (per 1 unit increase) | 2.27 (1.28-4.05) | 0.005 |
| LDL-C (per 1 unit increase) | 1.06 (0.95-2.55) | 0.057 |
| HDL-C (per 1 unit increase) | 0.94 (0.29-3.06) | 0.914 |
| eGFR (per 1 unit increase) | 1.00 (0.98-1.02) | 0.960 |
| Hs-CRP (per 1 unit increase) | 0.98 (0.84-1.14) | 0.801 |
| Multivessel disease (no as reference) | 0.92 (0.18-4.88) | 0.926 |
| Ostial lesion (no as reference) | 0.3 (0.06-1.37) | 0.12 |
| Bifurcation (no as reference) | 1.62 (0.59-4.49) | 0.349 |
| LM (no as reference) | - (0-Inf) | 0.998 |
| LAD (no as reference) | 0.12 (0.01-1.04) | 0.054 |
| LCX (no as reference) | 0.07 (0.01-0.93) | 0.044 |
| RCA (no as reference) | 0.12 (0.01-1.11) | 0.062 |
| Graft (no as reference) | 1.56 (0.05-47.01) | 0.799 |
| Number of lesions ≥ 2 (< 2 as reference) | 1.35 (0.44-4.09) | 0.6 |
| Number of stents ≥ 2 (< 2 as reference) | 0.71 (0.22-2.27) | 0.566 |
| Procedural success (failure as reference) | 0.76 (0.21-2.74) | 0.678 |
| DAPT (no as reference) | - (0-Inf) | 0.999 |
| Aspirin (no as reference) | 1.0 | 1.000 |
| Clopidogrel (no as reference) | 2.71 (0.22-32.78) | 0.433 |
| Ticagrelor (no as reference) | 2.66 (0.71-9.97) | 0.148 |
| β-blockers (no as reference) | 0.67 (0.06-7.2) | 0.742 |
| Statin (no as reference) | 1.19 (0.2-7.24) | 0.847 |
| Antidiabetic agents (no as reference) | 0.14 (0.03-0.63) | 0.01 |

Abbreviations: Tyg, triglyceride-glucose; CV, cardiovascular; TVMI, target vessel myocardial infarction; MACCEs, major adverse CV cerebral events; SHR, stress-hyperglycemia ratio; BMI, body mass index, T2DM, type 2 diabetes mellitus; HF, heart failure; AMI, acute myocardial infarction; PCI, percutaneous coronary intervention; CABG, coronary artery bypass grafting; PAD, peripheral artery disease; TC, total cholesterol; HDL-C, high-density lipoprotein cholesterol; LDL-C, low-density lipoprotein cholesterol; HbA1c, glycosylated hemoglobin A1c; FBG, fasting blood glucose; hs CRP, high-sensitivity C-reactive protein; eGFR, estimated glomerular filtration rate; LM, left main; DAPT, dual antiplatelet therapy.

**Table S2. MACCE risk according to baseline variables**

| Variables | HR (95%CI) | *P*-value |
| --- | --- | --- |
| Age (per one year increase) | 1.03 (1.01-1.06) | 0.003 |
| Male (female as reference) | 1.15 (0.61-2.18) | 0.669 |
| BMI (per 1 kg/m^2^ increase) | 0.96 (0.9-1.03) | 0.222 |
| Smoking (no as reference) | 0.91 (0.57-1.45) | 0.697 |
| Prediabetes (no as reference) | 0.76 (0.41-1.41) | 0.393 |
| Diabetes (no as reference) | 0.67 (0.31-1.45) | 0.310 |
| Hypertension (no as reference) | 1.9 (1.14-3.18) | 0.014 |
| Dyslipidemia (no as reference) | 0.83 (0.5-1.36) | 0.457 |
| HF (no as reference) | 2.75 (1.14-6.64) | 0.468 |
| Prior MI (no as reference) | 1.1 (0.7-1.73) | 0.673 |
| Prior stroke (no as reference) | 1.32 (0.81-2.16) | 0.263 |
| PAD (no as reference) | 1.42 (0.77-2.62) | 0.261 |
| Prior revascularization (no as reference) | 0.6 (0.12-3.11) | 0.545 |
| PCI (no as reference) | 2.13 (0.43-10.64) | 0.357 |
| CABG (no as reference) | 1.89 (0.57-6.32) | 0.298 |
| Renal dysfunction (no as reference) | 1.67 (0.89-3.13) | 0.108 |
| Unstable angina (no as reference) | 2.35 (0.27-20.43) | 0.439 |
| AMI (no as reference) | 0.78 (0.32-1.93) | 0.59 |
| Silent ischeamia (no as reference) | 2.59 (0.28-23.89) | 0.401 |
| Hemoglobin (per 1 unit increase) | 1.22 (0.8-1.87) | 0.35 |
| Platelet (per 1 unit increase) | 1 (1-1) | 0.649 |
| HbA1C (per 1 unit increase) | 0.94 (0.67-1.32) | 0.733 |
| FBG (per 1 unit increase) | 1.1 (0.95-1.28) | 0.22 |
| TC (per 1 unit increase) | 1 (0.46-2.2) | 0.996 |
| TG (per 1 unit increase) | 1.02 (0.79-1.32) | 0.87 |
| LDL-C (per 1 unit increase) | 1.03 (0.44-2.39) | 0.949 |
| HDL-C (per 1 unit increase) | 0.82 (0.45,1.51) | 0.526 |
| eGFR (per 1 unit increase) | 0.99 (0.99-1.00) | 1.107 |
| Hs-CRP (per 1 unit increase) | 1.06 (0.99-1.12) | 0.075 |
| Multivessel disease (no as reference) | 2.33 (1-5.43) | 0.051 |
| Ostial lesion (no as reference) | 0.9 (0.52-1.56) | 0.706 |
| Bifurcation (no as reference) | 0.98 (0.64-1.52) | 0.942 |
| LM (no as reference) | - (0-Inf) | 0.996 |
| LAD (no as reference) | 0.47 (0.15-1.49) | 0.199 |
| LCX (no as reference) | 0.43 (0.13-1.47) | 0.180 |
| RCA (no as reference) | 0.54 (0.17-1.68) | 0.286 |
| Graft (no as reference) | 0.97 (0.08-11.81) | 0.978 |
| Number of lesions ≥ 2 (< 2 as reference) | 0.89 (0.54-1.46) | 0.636 |
| Number of stents ≥ 2 (< 2 as reference) | 0.78 (0.48-1.26) | 0.308 |
| Procedural success (failure as reference) | 0.99 (0.56-1.75) | 0.970 |
| DAPT (no as reference) | 1.00 (0.13-1.23) | 0.997 |
| Aspirin (no as reference) |  |  |
| Clopidogrel (no as reference) | 1.26 (0.4-4.01) | 0.695 |
| Ticagrelor (no as reference) | 1.00 (0.53-1.88) | 0.998 |
| β-blockers (no as reference) | 0.92 (0.28-2.97) | 0.884 |
| Statin (no as reference) | 0.65 (0.33-1.27) | 0.210 |
| Antidiabetic agents (no as reference) | 1.02 (0.56-1.86) | 0.946 |

Abbreviations: Tyg, triglyceride-glucose; CV, cardiovascular; TVMI, target vessel myocardial infarction; MACCEs, major adverse CV cerebral events; SHR, stress-hyperglycemia ratio; BMI, body mass index, T2DM, type 2 diabetes mellitus; HF, heart failure; AMI, acute myocardial infarction; PCI, percutaneous coronary intervention; CABG, coronary artery bypass grafting; PAD, peripheral artery disease; TC, total cholesterol; HDL-C, high-density lipoprotein cholesterol; LDL-C, low-density lipoprotein cholesterol; HbA1c, glycosylated hemoglobin A1c; FBG, fasting blood glucose; hs CRP, high-sensitivity C-reactive protein; eGFR, estimated glomerular filtration rate; LM, left main; DAPT, dual antiplatelet therapy.

**Table S3. Baseline patient characteristics grouped by TyG index levels**

|  | All  (n=2740) | T1  (n=895) | T2  (n=917) | T3  (n=928) | *P*-value |
| --- | --- | --- | --- | --- | --- |
| Age, years | 58.5 ± 10.5 | 59.6 ± 10.6 | 58.6 ± 10.4 | 57.5 ± 10.4 | < 0.001 |
| Male, n (%) | 2272 (82.9) | 766 (85.6) | 754 (82.2) | 752 (81) | 0.028 |
| BMI, kg/m^2^ | 26.3 ± 3.3 | 25.6 ± 3.3 | 26.5 ± 3.3 | 26.8 ± 3.3 | < 0.001 |
| Smoking, n (%) | 880 (32.1) | 279 (31.2) | 286 (31.2) | 315 (33.9) | 0.341 |
| Prediabetes, n (%) | 999 (36.5) | 374 (41.8) | 416 (45.4) | 209 (22.5) | < 0.001 |
| Diabetes, n (%) | 1280 (46.7) | 302 (33.7) | 345 (37.6) | 633 (68.2) | < 0.001 |
| Hypertension, n (%) | 1777 (64.9) | 546 (61) | 616 (67.2) | 615 (66.3) | 0.012 |
| Dyslipidemia, n (%) | 2182 (79.6) | 673 (75.2) | 731 (79.7) | 778 (83.8) | < 0.001 |
| HF, n (%) | 211 (7.7) | 66 (7.4) | 66 (7.2) | 79 (8.5) | 0.690 |
| LVEF, % | 59.9 ± 8.7 | 59.9 ± 8.6 | 60.3 ± 8.4 | 59.4 ± 9.1 | 0.301 |
| Prior MI, n (%) | 901 (32.9) | 311 (34.7) | 285 (31.1) | 305 (32.9) | 0.251 |
| Prior stroke, n (%) | 350 (12.8) | 96 (10.7) | 130 (14.2) | 124 (13.4) | 0.072 |
| PAD, n (%) | 188 (6.9) | 59 (6.6) | 74 (8.1) | 55 (5.9) | 0.177 |
| Prior revascularization, n (%) | 870 (31.8) | 298 (33.3) | 274 (29.9) | 298 (32.1) | 0.283 |
| PCI | 822 (30.0) | 281 (31.4) | 256 (27.9) | 285 (30.7) | 0.229 |
| CABG | 86 (3.1) | 33 (3.7) | 31 (3.4) | 22 (2.4) | 0.239 |
| Renal dysfunction, n (%) | 129 (4.7) | 25 (2.8) | 44 (4.8) | 60 (6.5) | < 0.001 |
| Unstable angina, n (%) | 1162 (42.4) | 369 (41.2) | 402 (43.8) | 391 (42.1) | 0.520 |
| AMI, n (%) | 233 (8.5) | 73 (8.2) | 68 (7.4) | 92 (9.9) | 0.142 |
| Silent ischeamia, n (%) | 541 (19.7) | 184 (20.6) | 175 (19.1) | 182 (19.6) | 0.727 |
| Laboratory tests |  |  |  |  |  |
| Hemoglobin, g/L | 4.9 (4.5, 5.2) | 4.7 (4.4, 5.1) | 4.9 (4.5, 5.3) | 4.9 (4.6, 5.3) | < 0.001 |
| Platelet, ×10^9^/L | 222.0 (185.0, 261.2) | 214.0 (177.0, 251.0) | 225.0 (188.0, 266.8) | 226.0 (189.0, 267.0) | < 0.001 |
| HbA1C, % | 6.5 ± 1.2 | 6.2 ± 1.0 | 6.3 ± 1.0 | 7.0 ± 1.4 | < 0.001 |
| FBG, mmol/L | 6.6 ± 2.4 | 5.9 ± 2.0 | 5.9 ± 1.3 | 8.0 ± 3.0 | < 0.001 |
| TC, mmol/L | 3.8 (3.3, 4.6) | 3.5 (3.0, 4.0) | 3.9 (3.3, 4.5) | 4.2 (3.5, 5.1) | < 0.001 |
| TG, mmol/L | 1.4 (1.0, 2.0) | 0.8 (0.2, 1.0) | 1.5 (1.3, 1.7) | 2.3 (1.8, 3.0) | < 0.001 |
| LDL-C, mmol/L | 2.2 (1.8, 2.9) | 2.0 (1.6, 2.5) | 2.3 (1.8, 3.0) | 2.4 (1.8, 3.2) | < 0.001 |
| HDL-C, mmol/L | 1.0 (0.9, 1.2) | 1.1 (1.0, 1.3) | 1.0 (0.9, 1.2) | 1.0 (0.8, 1.1) | < 0.001 |
| eGFR, mL/min/1.73 m^2^ | 98.8 ± 26.2 | 100.4 ± 25.8 | 98.4 ± 25.8 | 97.9 ± 27.0 | 0.17 |
| Hs-CRP, mmol/L | 2.7 ± 3.2 | 2.5 ± 3.2 | 2.8 ± 3.2 | 2.8 ± 3.2 | 0.151 |
| TyG index | 8.8 ± 0.8 | 7.9 ± 0.7 | 8.8 ± 0.2 | 9.6 ± 0.4 | < 0.001 |
| SHR | 0.9 ± 0.2 | 0.8 ± 0.2 | 0.8 ± 0.1 | 0.9 ± 0.2 | < 0.001 |
| Angiographic characteristics, n (%) | | | | | |
| Multivessel disease | 2395 (87.4) | 778 (86.9) | 798 (87.0) | 819 (88.3) | 0.633 |
| Ostial lesion | 440 (16.1) | 138 (15.4) | 150 (16.4) | 152 (16.4) | 0.817 |
| Bifurcation | 944 (34.5) | 334 (37.3) | 315 (34.4) | 295 (31.8) | 0.046 |
| Intervention treatment, n (%) | | | | | |
| LM | 37 (1.4) | 11 (1.2) | 15 (1.6) | 11 (1.2) | 0.654 |
| LAD | 940 (34.3) | 337 (37.7) | 323 (35.2) | 280 (30.2) | 0.003 |
| LCX | 377 (13.8) | 122 (13.6) | 123 (13.4) | 132 (14.2) | 0.872 |
| RCA | 1367 (49.9) | 420 (46.9) | 459 (50.1) | 488 (52.6) | 0.054 |
| Graft | 7 (0.3) | 4 (0.4) | 3 (0.3) | 0 (0.0) | 0.094 |
| Number of lesions ≥ 2 | 732 (26.7) | 242 (27) | 241 (26.3) | 249 (26.8) | 0.931 |
| Number of stents ≥ 2 | 1546 (56.4) | 510 (57) | 512 (55.8) | 524 (56.5) | 0.885 |
| Procedural success | 2233 (81.5) | 739 (82.6) | 741 (80.8) | 753 (81.1) | 0.592 |
| Medication at discharge, n (%) | | | | | |
| DAPT | 2716 (99.1) | 887 (99.1) | 909 (99.1) | 920 (99.1) | 0.997 |
| Aspirin | 2740 (100.0) | 895 (100.0) | 917 (100.0) | 928 (100.0) | 1.000 |
| Clopidogrel | 2539 (92.7) | 829 (92.6) | 852 (92.9) | 858 (92.5) | 0.931 |
| Ticagrelor | 599 (21.9) | 200 (22.3) | 200 (21.8) | 199 (21.4) | 0.896 |
| β-blockers | 2509 (91.6) | 804 (89.8) | 836 (91.2) | 869 (93.6) | 0.012 |
| Statin | 2659 (97.0) | 868 (97.0) | 892 (97.3) | 899 (96.9) | 0.873 |
| Antidiabetic agents | 970 (35.4) | 234 (26.1) | 254 (27.7) | 482 (51.9) | < 0.001 |

TyG index tertiles: T1, < 8.56; T2 8.56-9.10; T3, ≥ 9.10.

Abbreviations: Tyg, triglyceride-glucose; CV, cardiovascular; TVMI, target vessel myocardial infarction; MACCEs, major adverse CV cerebral events; SHR, stress-hyperglycemia ratio; BMI, body mass index, T2DM, type 2 diabetes mellitus; HF, heart failure; LVEF, left ventricular ejection fraction; AMI, acute myocardial infarction; PCI, percutaneous coronary intervention; CABG, coronary artery bypass grafting; PAD, peripheral artery disease; TC, total cholesterol; HDL-C, high-density lipoprotein cholesterol; LDL-C, low-density lipoprotein cholesterol; HbA1c, glycosylated hemoglobin A1c; FBG, fasting blood glucose; hs CRP, high-sensitivity C-reactive protein; eGFR, estimated glomerular filtration rate; LM, left main; DAPT, dual antiplatelet therapy.

**Table S4. Baseline patient characteristics grouped by SHR levels**

|  | All  (n=2740) | T1  (n=897) | T2  (n=911) | T3  (n=932) | *P*-value |
| --- | --- | --- | --- | --- | --- |
| Age, years | 58.5 ± 10.5 | 58.9 ± 10.6 | 58.4 ± 10.7 | 58.4 ± 10.3 | 0.442 |
| Male, n (%) | 2272 (82.9) | 738 (82.3) | 756 (83.0) | 778 (83.5) | 0.790 |
| BMI, kg/m^2^ | 26.3 ± 3.3 | 26.2 ± 3.5 | 26.4 ± 3.4 | 26.3 ± 3.1 | 0.407 |
| Smoking, n (%) | 880 (32.1) | 309 (34.4) | 300 (32.9) | 271 (29.1) | 0.040 |
| Prediabetes, n (%) | 999 (36.5) | 393 (43.8) | 377 (41.4) | 229 (24.6) | < 0.001 |
| Diabetes, n (%) | 1280 (46.7) | 395 (44) | 296 (32.5) | 589 (63.2) | < 0.001 |
| Hypertension, n (%) | 1777 (64.9) | 578 (64.4) | 583 (64) | 616 (66.1) | 0.609 |
| Dyslipidemia, n (%) | 2182 (79.6) | 712 (79.4) | 731 (80.2) | 739 (79.3) | 0.856 |
| HF, n (%) | 211 (7.7) | 65 (7.2) | 59 (6.5) | 87 (9.3) | 0.084 |
| LVEF, % | 59.9 ± 8.7 | 59.8 ± 8.4 | 60.7 ± 8.4 | 59.2 ± 9.3 | 0.023 |
| Prior MI, n (%) | 901 (32.9) | 314 (35) | 278 (30.5) | 309 (33.2) | 0.124 |
| Prior stroke, n (%) | 350 (12.8) | 107 (11.9) | 114 (12.5) | 129 (13.8) | 0.453 |
| PAD, n (%) | 188 (6.9) | 65 (7.2) | 69 (7.6) | 54 (5.8) | 0.273 |
| Renal dysfunction, n (%) | 129 (4.7) | 36 (4.0) | 43 (4.7) | 50 (5.4) | < 0.001 |
| Prior revascularization, n (%) | 870 (31.8) | 301 (33.6) | 282 (31) | 287 (30.8) | 0.366 |
| PCI | 822 (30.0) | 279 (31.1) | 269 (29.5) | 274 (29.4) | 0.678 |
| CABG | 86 (3.1) | 35 (3.9) | 26 (2.9) | 25 (2.7) | 0.273 |
| Unstable angina, n (%) | 1162 (42.4) | 388 (43.3) | 375 (41.2) | 399 (42.8) | 0.637 |
| AMI, n (%) | 233 (8.5) | 71 (7.9) | 70 (7.7) | 92 (9.9) | 0.180 |
| Silent ischeamia, n (%) | 541 (19.7) | 162 (18.1) | 183 (20.1) | 196 (21.1) | 0.266 |
| Laboratory tests |  |  |  |  |  |
| Hemoglobin, g/L | 4.9 (4.5, 5.2) | 4.8 (4.5, 5.2) | 4.9 (4.5, 5.2) | 4.9 (4.5, 5.3) | 0.012 |
| Platelet, ×10^9^/L | 222.0 (185.0, 261.2) | 223.5 (188.0, 266.2) | 225.0 (188.0, 261.0) | 217.0 (180.5, 257.0) | 0.01 |
| HbA1C, % | 6.5 ± 1.2 | 6.7 ± 1.1 | 6.2 ± 1.0 | 6.7 ± 1.4 | < 0.001 |
| FBG, mmol/L | 6.6 ± 2.4 | 5.4 ± 1.1 | 6.0 ± 1.3 | 8.4 ± 3.0 | < 0.001 |
| TC, mmol/L | 3.8 (3.3, 4.6) | 3.8 (3.2, 4.5) | 3.9 (3.3, 4.7) | 3.8 (3.2, 4.6) | 0.077 |
| TG, mmol/L | 1.4 (1.0, 2.0) | 1.4 (1.0, 2.0) | 1.4 (1.0, 2.0) | 1.5 (1.0, 2.1) | 0.062 |
| LDL-C, mmol/L | 2.2 (1.8, 2.9) | 2.2 (1.7, 2.8) | 2.3 (1.8, 3.0) | 2.2 (1.7, 2.9) | 0.037 |
| HDL-C, mmol/L | 1.0 (0.9, 1.2) | 1.0 (0.9, 1.2) | 1.0 (0.9, 1.3) | 1.0 (0.9, 1.2) | 0.191 |
| eGFR, mL/min/1.73 m^2^ | 98.8 ± 26.2 | 98.2 ± 26.2 | 99.5 ± 26.0 | 98.8 ± 26.5 | 0.633 |
| Hs-CRP, mmol/L | 1.5 (0.7, 3.2) | 1.5 (0.7, 3.3) | 1.4 (0.7, 3.0) | 1.4 (0.7, 3.3) | 0.532 |
| TyG index | 8.8 ± 0.8 | 8.5 ± 0.8 | 8.7 ± 0.7 | 9.0 ± 0.9 | < 0.001 |
| SHR | 0.9 ± 0.2 | 0.7 ± 0.1 | 0.8 ± 0.0 | 1.0 ± 0.2 | < 0.001 |
| Angiographic characteristics, n (%) | | | | | |
| Multivessel disease | 2395 (87.4) | 780 (87.0) | 792 (86.9) | 823 (88.3) | 0.597 |
| Ostial lesion | 440 (16.1) | 135 (15.1) | 146 (16.0) | 159 (17.1) | 0.504 |
| Bifurcation | 944 (34.5) | 302 (33.7) | 324 (35.6) | 318 (34.1) | 0.674 |
| Intervention treatment, n (%) | | | | | |
| LM | 37 (1.4) | 9 (1.0) | 13 (1.4) | 15 (1.6) | 0.517 |
| LAD | 940 (34.3) | 306 (34.1) | 322 (35.3) | 312 (33.5) | 0.692 |
| LCX | 377 (13.8) | 122 (13.6) | 117 (12.8) | 138 (14.8) | 0.466 |
| RCA | 1367 (49.9) | 455 (50.7) | 452 (49.6) | 460 (49.4) | 0.825 |
| Graft | 7 (0.3) | 1 (0.1) | 1 (0.1) | 5 (0.5) | 0.229 |
| Number of lesions ≥ 2 | 732 (26.7) | 228 (25.4) | 232 (25.5) | 272 (29.2) | 0.111 |
| Number of stents ≥ 2 | 1546 (56.4) | 493 (55) | 527 (57.8) | 526 (56.4) | 0.465 |
| Procedural success | 2233 (81.5) | 728 (81.2) | 747 (82) | 758 (81.3) | 0.888 |
| Medication at discharge, n (%) | | | | | |
| DAPT | 2716 (99.1) | 887 (98.9) | 905 (99.3) | 924 (99.1) | 0.580 |
| Aspirin | 2740 (100.0) | 897 (100.0) | 911 (100.0) | 932 (100.0) | 1.000 |
| Clopidogrel | 2539 (92.7) | 824 (91.9) | 849 (93.2) | 866 (92.9) | 0.518 |
| Ticagrelor | 599 (21.9) | 212 (23.6) | 184 (20.2) | 203 (21.8) | 0.209 |
| β-blockers | 2509 (91.6) | 816 (91) | 822 (90.2) | 871 (93.5) | 0.033 |
| Statin | 2659 (97.0) | 871 (97.1) | 887 (97.4) | 901 (96.7) | 0.676 |
| Antidiabetic agents | 970 (35.4) | 275 (30.7) | 241 (26.5) | 454 (48.7) | < 0.001 |

SHR tertiles: T1, < 0.76; T2 0.76-0.86; T3, ≥ 0.86.

Abbreviations: Tyg, triglyceride-glucose; CV, cardiovascular; TVMI, target vessel myocardial infarction; MACCEs, major adverse CV cerebral events; SHR, stress-hyperglycemia ratio; BMI, body mass index, T2DM, type 2 diabetes mellitus; HF, heart failure; LVEF, left ventricular ejection fraction; AMI, acute myocardial infarction; PCI, percutaneous coronary intervention; CABG, coronary artery bypass grafting; PAD, peripheral artery disease; TC, total cholesterol; HDL-C, high-density lipoprotein cholesterol; LDL-C, low-density lipoprotein cholesterol; HbA1c, glycosylated hemoglobin A1c; FBG, fasting blood glucose; hs CRP, high-sensitivity C-reactive protein; eGFR, estimated glomerular filtration rate; LM, left main; DAPT, dual antiplatelet therapy.

**Table S5. Survival analysis for the association of the TyG index and SHR with the risk of all-cause death**

|  | Per one unit increase^#^ | Groups | | | *P* for trend |
| --- | --- | --- | --- | --- | --- |
|  |  | T1 | T2 | T3 |  |
| **TyG index** | | | | | |
| No./Subject |  | 22/895 | 22/917 | 36/928 |  |
| Crude | 1.38 (1.04-1.83) | 1.00 | 0.96 (0.53-1.74) | 1.61 (0.95-2.74) | 0.063 |
| Model 1 | 1.68 (1.24-2.29) | 1.00 | 1.18 (0.65-2.14) | 2.26 (1.31-3.91) | 0.003 |
| Model 2 | 1.71 (1.23-2.37) | 1.00 | 1.19 (0.65-2.18) | 2.30 (1.29-4.11) | 0.004 |
| Model 3 | 1.70 (1.17-2.48) | 1.00 | 1.14 (0.62-2.07) | 2.22 (1.24-3.95) | 0.006 |
| **SHR** | | | | | |
| No./Subject |  | 14/897 | 23/911 | 43/932 |  |
| Crude | 1.27 (1.17-1.38) | 1.00 | 1.59 (0.82-3.1) | 3.02 (1.65-5.52) | < 0.001 |
| Model 1 | 1.26 (1.16-1.37) | 1.00 | 1.69 (0.87-3.28) | 3.36 (1.84-6.14) | < 0.001 |
| Model 2 | 1.27 (1.16-1.39) | 1.00 | 1.67 (0.85-3.27) | 3.20 (1.72-5.93) | < 0.001 |
| Model 3 | 1.21 (1.09-1.35) | 1.00 | 1.63 (0.79-3.36) | 2.79 (1.44-5.42) | 0.002 |

Model 1: adjusted for age, sex, and BMI.

Model 2: adjusted for age, sex, BMI, smoking, T2DM, dyslipidemia, hypertension, prior HF, prior MI, prior stroke, peripheral vascular disease, prior revascularization, and AMI.

Model 3: adjusted for age, sex, BMI, smoking, HF, T2DM, dyslipidemia, hypertension, prior MI, prior stroke, peripheral vascular disease, prior revascularization, AMI, multivessel disease, ostial lesion, bifurcation, number of lesions ≥ 2, number of stents ≥ 2, procedural success, eGFR, DAPT, statin, and anti-diabetic drug.

^#^ Per one unit refers to “1.00” in the Tyg index and “0.10” in SHR.

Abbreviations: TyG, triglyceride-glucose; CV, cardiovascular; eGFR, estimated glomerular filtration rate; MI, myocardial infarction; MACCEs, major adverse cardiovascular cerebral events; SHR, stress-hyperglycemia ratio; BMI, body mass index, T2DM, type 2 diabetes mellitus; HF, heart failure; AMI, acute myocardial infarction; DAPT, dual anti-platelet therapy.

**Table S6. Correlation analysis between the TyG index/SHR and** **TC, LDL-C, and hs-CRP**

| Variables | R^2^ (95%CI) | *P*-value |
| --- | --- | --- |
| TyG index |  |  |
| TC, mmol/L | 0.64 (0.56-0.73) | <0.001 |
| LDL-C, mmol/L | 0.25 (0.18-0.33) | <0.001 |
| Hs-CRP, mmol/L | 0.07 (-0.23-0.37) | 0.670 |
| SHR |  |  |
| TC, mmol/L | 0.30 (0.04-0.56) | 0.025 |
| LDL-C, mmol/L | 0.17 (-0.06-0.40) | 0.144 |
| Hs-CRP, mmol/L | 0.94 (0.05-1.82) | 0.038 |

The Cox model is adjusted with age, sex, BMI, smoking, HF, T2DM, dyslipidemia, hypertension, prior MI, prior stroke, peripheral vascular disease, prior revascularization, AMI, multivessel disease, ostial lesion, bifurcation, number of lesions ≥ 2, number of stents ≥ 2, procedural success, eGFR, DAPT, statin, and anti-diabetic drug.

Abbreviations: TyG, triglyceride-glucose; CV, cardiovascular; eGFR, estimated glomerular filtration rate; MI, myocardial infarction; MACCEs, major adverse cardiovascular cerebral events; SHR, stress-hyperglycemia ratio; BMI, body mass index, T2DM, type 2 diabetes mellitus; HF, heart failure; AMI, acute myocardial infarction; DAPT, dual anti-platelet therapy.

**Table S7. Cox regression models for the association of TyG index and SHR with clinical outcomes in patients with different glucose statuses**

|  | Per one unit increase^#^ | Groups | | | *P* for trend |
| --- | --- | --- | --- | --- | --- |
|  |  | T1 | T2 | T3 |  |
| **NG** |  |  |  |  |  |
| TyG index |  |  |  |  |  |
| CV death and TVMI | 3.73 (0.85-16.49) | 1.00 | 5.67 (0.53-61.09) | 6.62 (0.52-84.13) | 0.141 |
| MACCEs | 1.51 (0.67-3.42) | 1.00 | 3.14 (0.80-12.28) | 2.20 (0.47-10.23) | 0.397 |
| SHR |  |  |  |  |  |
| CV death and TVMI | 1.66 (1.18-2.35) | 1.00 | 0.51 (0.04-6.62) | 1.81 (0.25-13.11) | 0.519 |
| MACCEs | 1.41 (0.99-2.02) | 1.00 | 1.57 (0.46-5.30) | 1.35 (0.38-4.75) | 0.675 |
| **Pre-DM** |  |  |  |  |  |
| TyG index |  |  |  |  |  |
| CV death and TVMI | 0.87 (0.32-2.35) | 1.00 | 0.53 (0.08-3.57) | 1.99 (0.41-9.63) | 0.344 |
| MACCEs | 1.31 (0.80-2.14) | 1.00 | 1.07 (0.47-2.46) | 1.67 (0.77-3.59) | 0.171 |
| SHR |  |  |  |  |  |
| CV death and TVMI | 2.53 (1.35-4.77) | 1.00 | 4.81 (0.49-47.39) | 17.7 (1.82-172.27) | 0.007 |
| MACCEs | 1.39 (1.05-1.83) | 1.00 | 1.30 (0.54-3.08) | 2.91 (1.32-6.43) | 0.006 |
| **T2DM** |  |  |  |  |  |
| TyG index |  |  |  |  |  |
| CV death and TVMI | 2.26 (1.13-4.52) | 1.00 | 5.18 (0.9-29.94) | 10.51 (1.79-61.63) | 0.006 |
| MACCEs | 2.12 (1.5-2.99) | 1.00 | 2.84 (1.42-5.69) | 3.11 (1.54-6.32) | 0.002 |
| SHR |  |  |  |  |  |
| CV death and TVMI | 1.22 (1.06-1.39) | 1.00 | 2.7 (0.67-10.93) | 4.33 (1.18-15.92) | 0.022 |
| MACCEs | 1.21 (1.12-1.31) | 1.00 | 3.21 (1.55-6.66) | 3.88 (1.91-7.88) | <0.001 |

The Cox model is adjusted with age, sex, BMI, smoking, HF, T2DM, dyslipidemia, hypertension, prior MI, prior stroke, peripheral vascular disease, prior revascularization, AMI, multivessel disease, ostial lesion, bifurcation, number of lesions ≥ 2, number of stents ≥ 2, procedural success, eGFR, DAPT, statin, and anti-diabetic drug.

NG TyG: T1, < 8.37; T2, 8.37-8.82; T3, > 8.82. NG SHR: T1, < 0.78; T2, 0.78-0.85; T3, > 0.85.

Pre-DM TyG: T1, < 8.51; T2, 8.51-8.92; T3, > 8.92. Pre-DM SHR: T1, < 0.75; T2, 0.75-0.82; T3, > 0.82.

T2DM TyG: T1, < 9.34; T2, 8.80-9.33; T3, > 9.33. T2DM SHR: T1, <0.78; T2, 0.78-0.94; T3, > 0.94.

^#^ Per one unit refers to “1.00” in the Tyg index and “0.10” in SHR.

***P*-interaction**: 1) CV death and TVMI: *P* Tyg, 0.066; *P* SHR,0.527; 2) MACCEs: *P* Tyg, 0.045; *P* SHR, 0.458.

Abbreviations: TyG, triglyceride-glucose; CV, cardiovascular; eGFR, estimated glomerular filtration rate; MI, myocardial infarction; MACCEs, major adverse cardiovascular cerebral events; SHR, stress-hyperglycemia ratio; BMI, body mass index, T2DM, type 2 diabetes mellitus; HF, heart failure; AMI, acute myocardial infarction; DAPT, dual anti-platelet therapy.

**Table S8. Cox regression models for the association of TyG index and SHR with clinical outcomes in patients with different procedural outcomes**

|  | Per one unit increase^#^ | Groups | | | *P* for trend |
| --- | --- | --- | --- | --- | --- |
|  |  | T1 | T2 | T3 |  |
| **Procedural success** |  |  |  |  |  |
| TyG index |  |  |  |  |  |
| CV death and TVMI | 1.74 (1.01-3.01) | 1.00 | 1.28 (0.43-3.87) | 3.79 (1.27-11.28) | 0.012 |
| MACCEs | 1.65 (1.24-2.20) | 1.00 | 1.18 (0.68-2.03) | 2.25 (1.31-3.85) | 0.002 |
| SHR |  |  |  |  |  |
| CV death and TVMI | 1.22 (1.05-1.42) | 1.00 | 2.60 (0.77-8.78) | 4.89 (1.55-15.41) | 0.005 |
| MACCEs | 1.22 (1.12-1.33) | 1.00 | 1.53 (0.92-2.55) | 2.69 (1.68-4.31) | <0.001 |
| **Procedural failure** |  |  |  |  |  |
| TyG index |  |  |  |  |  |
| CV death and TVMI | 4.99 (1.01-24.61) | 1.00 | 3.11 (0.18-54.52) | 14.25 (1.05-192.93) | 0.018 |
| MACCEs | 3.70 (1.80-7.62) | 1.00 | 2.29 (0.78-6.69) | 3.86 (1.38-10.78) | 0.008 |
| SHR |  |  |  |  |  |
| CV death and TVMI | 1.55 (1.12-2.16) | 1.00 | 8.26 (0.67-101.45) | 10.65 (1.08-105.28) | 0.039 |
| MACCEs | 1.27 (1.08-1.49) | 1.00 | 2.77 (0.94-8.18) | 3.74 (1.35-10.39) | 0.010 |

The Cox model is adjusted with age, sex, BMI, smoking, HF, T2DM, dyslipidemia, hypertension, prior MI, prior stroke, peripheral vascular disease, prior revascularization, AMI, multivessel disease, ostial lesion, bifurcation, number of lesions ≥ 2, number of stents ≥ 2, procedural success, eGFR, DAPT, statin, and anti-diabetic drug.

PS tyg: T1, < 8.56; T2, 8.56-9.10; T3, > 9.10. PS shr: T1, < 0.76; T2, 0.76-0.87; T3, > 0.87.

PF tyg: T1, < 8.60; T2, 8.60-9.11; T3, > 9.11. PF shr: T1, < 0.78; T2, 0.78-0.87; T3, > 0.87.

# Per one unit refers to “1.00” in the Tyg index and “0.10” in SHR.

***P*-interaction**: 1) CV death and TVMI: P Tyg, 0.249; P SHR, 0.730; 2) MACCEs: P Tyg, 0.168; P SHR, 0.524.

Abbreviations: TyG, triglyceride-glucose; CV, cardiovascular; eGFR, estimated glomerular filtration rate; MI, myocardial infarction; MACCEs, major adverse cardiovascular cerebral events; SHR, stress-hyperglycemia ratio; BMI, body mass index, T2DM, type 2 diabetes mellitus; HF, heart failure; AMI, acute myocardial infarction; DAPT, dual anti-platelet therapy.

**Table S9. Survival analysis for the association of the combination of Tyg index and SHR with the risk of all-cause death**

|  | Groups | | | | *P* for trend |
| --- | --- | --- | --- | --- | --- |
|  | Low TyG and  low SHR | Low TyG and  high SHR | High TyG and  low SHR | High TyG and  high SHR |  |
| No./Subject | 30/1353 | 14/460 | 7/455 | 29/472 |  |
| Crude | 1.00 | 1.35 (0.71-2.54) | 0.68 (0.30-1.55) | 2.97 (1.78-4.95) | 0.001 |
| Model 1 | 1.00 | 1.46 (0.78-2.76) | 0.91 (0.40-2.09) | 3.70 (2.20-6.22) | < 0.001 |
| Model 2 | 1.00 | 1.46 (0.73-2.94) | 0.90 (0.36-2.25) | 2.87 (1.51-5.46) | 0.006 |
| Model 3 | 1.00 | 1.50 (0.73-3.05) | 0.92 (0.37-2.30) | 2.93 (1.53-5.60) | 0.005 |

Model 1: adjusted for age, sex, and BMI.

Model 2: adjusted for age, sex, BMI, smoking, T2DM, dyslipidemia, hypertension, prior HF, prior MI, prior stroke, peripheral vascular disease, prior revascularization, and AMI.

Model 3: age, sex, BMI, smoking, HF, T2DM, dyslipidemia, hypertension, prior MI, prior stroke, peripheral vascular disease, prior revascularization, AMI, multivessel disease, ostial lesion, bifurcation, number of lesions ≥ 2, number of stents ≥ 2, procedural success, eGFR, DAPT, statin, and anti-diabetic drug.

Low TyG index or SHR: T1-2; High Tyg index or SHR: T3

**Table S10. Subgroup analyses of the associations between TyG index and the risk of the composite of CV death and target vessel MI**

| Subgroup | | No. case/Total | Groups | | | *P* for interaction |
| --- | --- | --- | --- | --- | --- | --- |
|  |  |  | T1 | T2 | T3 |  |
| Age (years) | |  |  |  |  | 0.749 |
| <60 | | 13/1436 | 1.00 | 1.90 (0.16-21.96) | 4.64 (0.41-52.5) |  |
| ≥60 | | 34/1304 | 1.00 | 1.48 (0.45-4.86) | 4.97 (1.53-16.12) |  |
| Sex | |  |  |  |  | 0.006 |
| Male | | 33/2272 | 1.00 | 4.59 (0.98-21.4) | 12.19 (2.59-57.44) |  |
| Female | | 14/468 | 1.00 | 0.30 (0.06-1.53) | 1.03 (0.33-3.23) |  |
| BMI, kg/m^2^ | |  |  |  |  | 0.997 |
| <30 | | 43/2395 | 1.00 | 1.40 (0.49-3.97) | 4.23 (1.53-11.68) |  |
| ≥30 | | 4/345 | 1.00 | 0.68 (0-Inf) | 1.12 (0-Inf) |  |
| Smoking | |  |  |  |  | 0.550 |
| Yes | | 35/1860 | 1.00 | 1.90 (0.68-5.28) | 5.17 (1.86-14.36) |  |
| No | | 12/880 | 1.00 | 1.32 (0.11-15.35) | 5.91 (0.68-51.40) |  |
| Hyperlipidemia | |  |  |  |  | 0.063 |
| Yes | | 39/2182 | 1.00 | 0.88 (0.29-2.68) | 3.90 (1.42-10.71) |  |
| No | | 5/588 | 1.00 | 7.0e+10 (0-Inf) | 7.6e+10 (0-Inf) |  |
| Hypertension | |  |  |  |  | 0.325 |
| Yes | | 39/1777 | 1.00 | 1.14 (0.39-3.29) | 3.80 (1.37-10.54) |  |
| No | | 8/963 | 1.00 | 2.2e+9 (0-Inf) | 3.8e+9 (0-Inf) |  |
| HF with LVEF < 50 % | |  |  |  |  | 0.424 |
| Yes | | 12/194 | 1.00 | 0.33 (0.03-3.2) | 4.15 (0.72-23.9) |  |
| No | | 35/2546 | 1.00 | 1.94 (0.59-6.45) | 5.18 (1.61-16.66) |  |
| Renal dysfunction | |  |  |  |  | 0.201 |
| Yes | | 14/129 | 1.00 | 0.65 (0.07-5.87) | 1.48 (0.14-16.16) |  |
| No | | 33/2611 | 1.00 | 1.96 (0.49-7.87) | 6.41 (1.69-24.26) |  |
| AMI |  | |  |  |  | 0.081 |
| Yes | | 6/233 | 1.00 | 3.25e+10 (0-Inf) | 1.90e+10 (0-Inf) |  |
| No | | 41/2507 | 1.00 | 1.49 (0.54-4.14) | 3.40 (1.21-9.5) |  |

The Cox model is adjusted with age, sex, BMI, smoking, HF, T2DM, dyslipidemia, hypertension, prior MI, prior stroke, peripheral vascular disease, prior revascularization, AMI, multivessel disease, ostial lesion, bifurcation, number of lesions ≥ 2, number of stents ≥ 2, procedural success, eGFR, DAPT, statin, and anti-diabetic drug.

Abbreviations: TyG, triglyceride-glucose; CV, cardiovascular; eGFR, estimated glomerular filtration rate; MI, myocardial infarction; MACCEs, major adverse cardiovascular cerebral events; SHR, stress-hyperglycemia ratio; BMI, body mass index, T2DM, type 2 diabetes mellitus; HF, heart failure; LVEF, left ventricular ejection fraction; AMI, acute myocardial infarction; DAPT, dual anti-platelet therapy.

**Table S11. Subgroup analyses of the associations between TyG index and the risk of MACCEs**

| Subgroup | No. case/Total | Groups | | | *P* for interaction |
| --- | --- | --- | --- | --- | --- |
|  |  | T1 | T2 | T3 |  |
| Age (years) |  |  |  |  | 0.665 |
| <60 | 66/1436 | 1.00 | 0.89 (0.38-2.11) | 1.93 (0.89-4.22) |  |
| ≥60 | 103/1304 | 1.00 | 1.66 (0.91-3.03) | 2.88 (1.57-5.30) |  |
| Sex |  |  |  |  | 0.401 |
| Male | 133/2272 | 1.00 | 1.4 (0.81-2.43) | 2.99 (1.75-5.13) |  |
| Female | 36/468 | 1.00 | 0.90 (0.30-2.68) | 1.52 (0.49-4.68) |  |
| BMI, kg/m^2^ |  |  |  |  | 0.972 |
| <30 | 153/2395 | 1.00 | 1.32 (0.80-2.20) | 2.53 (1.54-4.17) |  |
| ≥30 | 16/345 | 1.00 | 1.40 (0.22-8.74) | 2.28 (0.35-14.87) |  |
| Smoking |  |  |  |  | 0.345 |
| Yes | 54/880 | 1.00 | 0.76 (0.31-1.84) | 1.95 (0.88-4.33) |  |
| No | 115/1860 | 1.00 | 1.69 (0.93-3.08) | 2.86 (1.56-5.23) |  |
| Hyperlipidemia |  |  |  |  | 0.739 |
| Yes | 136/2182 | 1.00 | 1.22 (0.70-2.14) | 2.48 (1.45-4.24) |  |
| No | 33/558 | 1.00 | 1.48 (0.51-4.28) | 2.78 (0.90-8.57) |  |
| Hypertension |  |  |  |  | 0.261 |
| Yes | 134/1777 | 1.00 | 1.15 (0.67-1.99) | 2.49 (1.48-4.20) |  |
| No | 35/963 | 1.00 | 1.52 (0.50-4.62) | 2.36 (0.72-7.72) |  |
| HF with LVEF < 50 % |  |  |  |  | 0.136 |
| Yes | 23/194 | 1.00 | 1.27 (0.13-12.43) | 10.79 (1.30-89.49) |  |
| No | 146/2546 | 1.00 | 1.41 (0.85-2.34) | 2.34 (1.41-3.89) |  |
| Renal dysfunction |  |  |  |  | 0.226 |
| Yes | 21/129 | 1.00 | 0.42 (0.07-2.37) | 1.44 (0.28-7.38) |  |
| No | 148/2611 | 1.00 | 1.52 (0.90-2.58) | 2.65 (1.57-4.45) |  |
| AMI | |  |  |  | 0.030 |
| Yes | 13/233 | 1.00 | 1.08 (0.70-1.67) | 2.13 (1.41-3.23) |  |
| No | 156/2507 | 1.00 | 1.38 (0.31-6.04) | 3.31 (0.97-11.29) |  |

The Cox model is adjusted with age, sex, BMI, smoking, HF, T2DM, dyslipidemia, hypertension, prior MI, prior stroke, peripheral vascular disease, prior revascularization, AMI, multivessel disease, ostial lesion, bifurcation, number of lesions ≥ 2, number of stents ≥ 2, procedural success, eGFR, DAPT, statin, and anti-diabetic drug.

Abbreviations: TyG, triglyceride-glucose; CV, cardiovascular; eGFR, estimated glomerular filtration rate; MI, myocardial infarction; MACCEs, major adverse cardiovascular cerebral events; SHR, stress-hyperglycemia ratio; BMI, body mass index, T2DM, type 2 diabetes mellitus; HF, heart failure; LVEF, left ventricular ejection fraction; AMI, acute myocardial infarction; DAPT, dual anti-platelet therapy.

**Table S12. Subgroup analyses of the associations between SHR and the risk of the composite of CV death and target vessel MI**

| Subgroup | No. case/Total | Groups | | | *P* for interaction |
| --- | --- | --- | --- | --- | --- |
|  |  | T1 | T2 | T3 |  |
| Age (years) |  |  |  |  | 0.096 |
| <60 | 13/1436 | 1.00 | 1.15 (0.21-6.28) | 1.35 (0.25-7.20) |  |
| ≥60 | 34/1304 | 1.00 | 6.53 (1.33-32.19) | 15.72 (3.42-72.14) |  |
| Sex |  |  |  |  | 0.081 |
| Male | 33/2272 | 1.00 | 2.29 (0.75-7.04) | 3.34 (1.15-9.67) |  |
| Female | 14/468 | 1.00 | 1.31e+9 (0-Inf) | 1.24e+9 (0-Inf) |  |
| BMI, kg/m^2^ |  |  |  |  | 0.061 |
| <30 | 43/2395 | 1.00 | 2.79 (0.84-9.26) | 7.09 (2.35-21.37) |  |
| ≥30 | 4/345 | 1.00 | 1.25e+4 (0-Inf) | 0 (0-Inf) |  |
| Smoking |  |  |  |  | 0.236 |
| Yes | 35/1860 | 1.00 | 2.86 (0.49-16.82) | 2.73 (0.42-17.84) |  |
| No | 12/880 | 1.00 | 3.34 (0.79-14.13) | 10.13 (2.77-37.1) |  |
| Hyperlipidemia |  |  |  |  | 0.091 |
| Yes | 39/2182 | 1.00 | 3.79 (1.04-13.82) | 7.35 (2.11-25.55) |  |
| No | 5/588 | 1.00 | 0 (0-Inf) | 1.67 (0.28-10.02) |  |
| Hypertension |  |  |  |  | 0.182 |
| Yes | 39/1777 | 1.00 | 3.95 (1.08-14.36) | 7.32 (2.11-25.34) |  |
| No | 8/963 | 1.00 | 0.49 (0.04-5.45) | 2.68 (0.52-13.81) |  |
| HF with LVEF < 50 % |  |  |  |  | 0.813 |
| Yes | 12/194 | 1.00 | 14.74 (0.38-568.13) | 113.39 (2.76-4666.44) |  |
| No | 35/2546 | 1.00 | 3.58 (1.13-11.31) | 6.34 (2.09-19.23) |  |
| Renal dysfunction |  |  |  |  | 0.647 |
| Yes | 14/129 | 1.00 | 94.77 (1.91-4711.34) | 116.95 (3.14-4351.24) |  |
| No | 33/2611 | 1.00 | 2.37 (0.73-7.71) | 5.34 (1.78-16.03) |  |
| AMI | |  |  |  | 0.479 |
| Yes | 6/233 | 1.00 | 0 (0-Inf) | 9.58e+32 (0-Inf) |  |
| No | 41/2507 | 1.00 | 2.75 (0.98-7.73) | 5.04 (1.87-13.59) |  |

The Cox model is adjusted with age, sex, BMI, smoking, HF, T2DM, dyslipidemia, hypertension, prior MI, prior stroke, peripheral vascular disease, prior revascularization, AMI, multivessel disease, ostial lesion, bifurcation, number of lesions ≥ 2, number of stents ≥ 2, procedural success, eGFR, DAPT, statin, and anti-diabetic drug.

Abbreviations: TyG, triglyceride-glucose; CV, cardiovascular; eGFR, estimated glomerular filtration rate; MI, myocardial infarction; MACCEs, major adverse cardiovascular cerebral events; SHR, stress-hyperglycemia ratio; BMI, body mass index, T2DM, type 2 diabetes mellitus; HF, heart failure; LVEF, left ventricular ejection fraction; AMI, acute myocardial infarction; DAPT, dual anti-platelet therapy.

**Table S13. Subgroup analyses of the associations between SHR and the risk of MACCEs**

| Subgroup | No. case/Total | Groups | | | P for interaction |
| --- | --- | --- | --- | --- | --- |
|  |  | T1 | T2 | T3 |  |
| Age (years) |  |  |  |  | 0.287 |
| <60 | 66/1436 | 1.00 | 0.92 (0.42-2.05) | 1.86 (0.91-3.77) |  |
| ≥60 | 103/1304 | 1.00 | 2.17 (1.13-4.19) | 3.83 (2.06-7.11) |  |
| Sex |  |  |  |  | 0.497 |
| Male | 133/2272 | 1.00 | 1.4 (0.80-2.44) | 2.64 (1.59-4.38) |  |
| Female | 36/468 | 1.00 | 3.47 (0.97-12.35) | 4.19 (1.22-14.36) |  |
| BMI, kg/m^2^ |  |  |  |  | 0.793 |
| <30 | 153/2395 | 1.00 | 1.51 (0.90-2.56) | 2.91 (1.80-4.70) |  |
| ≥30 | 16/345 | 1.00 | 2.37 (0.37-15.18) | 3.67 (0.61-22.21) |  |
| Smoking |  |  |  |  | 0.486 |
| Yes | 54/880 | 1.00 | 1.06 (0.45-2.54) | 2.95 (1.34-6.49) |  |
| No | 115/1860 | 1.00 | 1.9 (1.01-3.56) | 3.26 (1.80-5.90) |  |
| Hyperlipidemia |  |  |  |  | 0.741 |
| Yes | 136/2182 | 1.00 | 1.71 (0.98-3.00) | 3.02 (1.79-5.08) |  |
| No | 33/558 | 1.00 | 1.35 (0.40-4.56) | 3.85 (1.29-11.43) |  |
| Hypertension |  |  |  |  | 0.689 |
| Yes | 134/1777 | 1.00 | 1.75 (1.00-3.08) | 3.13 (1.87-5.26) |  |
| No | 35/963 | 1.00 | 0.88 (0.28-2.75) | 1.46 (0.51-4.19) |  |
| HF with LVEF < 50 % |  |  |  |  | 0.146 |
| Yes | 23/194 | 1.00 | 1.23 (0.09-17.21) | 8.69 (0.91-82.99) |  |
| No | 146/2546 | 1.00 | 1.56 (0.93-2.61) | 2.64 (1.64-4.27) |  |
| Renal dysfunction |  |  |  |  | 0.955 |
| Yes | 21/129 | 1.00 | 2.57 (0.51-12.97) | 6.2 (1.27-30.22) |  |
| No | 148/2611 | 1.00 | 1.51 (0.89-2.58) | 2.84 (1.74-4.64) |  |
| AMI | |  |  |  | 0.099 |
| Yes | 13/233 | 1.00 | 1.43 (0.90-2.26) | 2.8 (1.83-4.27) |  |
| No | 156/2507 | 1.00 | 1.44 (0.38-5.52) | 2.32 (0.71-7.58) |  |

The Cox model is adjusted with age, sex, BMI, smoking, HF, T2DM, dyslipidemia, hypertension, prior MI, prior stroke, peripheral vascular disease, prior revascularization, AMI, multivessel disease, ostial lesion, bifurcation, number of lesions ≥ 2, number of stents ≥ 2, procedural success, eGFR, DAPT, statin, and anti-diabetic drug.

Abbreviations: TyG, triglyceride-glucose; CV, cardiovascular; eGFR, estimated glomerular filtration rate; MI, myocardial infarction; MACCEs, major adverse cardiovascular cerebral events; SHR, stress-hyperglycemia ratio; BMI, body mass index, T2DM, type 2 diabetes mellitus; HF, heart failure; LVEF, left ventricular ejection fraction; AMI, acute myocardial infarction; DAPT, dual anti-platelet therapy.

**Table S14. Sensitivity analyses** **of the associations of TyG index and SHR with outcomes after excluding patients** **who presented clinical events within 90 days (n=2724)**

| Analysis | Categories | | | *P* for trend |
| --- | --- | --- | --- | --- |
|  | T1 | T2 | T3 |  |
| TyG index |  |  |  |  |
| CV death and target vessel MI | 1.00 | 1.50 (0.54-4.15) | 4.21 (1.55-11.41) | 0.002 |
| MACCEs | 1.00 | 1.35 (0.82-2.21) | 2.36 (1.45-3.85) | <0.001 |
| SHR |  |  |  |  |
| CV death and target vessel MI | 1.00 | 1.63 (0.98-2.71) | 2.96 (1.85-4.73) | <0.001 |
| MACCEs | 1.00 | 1.35 (0.82-2.21) | 2.36 (1.45-3.85) | <0.001 |

The Cox model is adjusted with age, sex, BMI, smoking, HF, T2DM, dyslipidemia, hypertension, prior MI, prior stroke, peripheral vascular disease, prior revascularization, AMI, multivessel disease, ostial lesion, bifurcation, number of lesions ≥ 2, number of stents ≥ 2, procedural success, eGFR, DAPT, statin, and anti-diabetic drug.

Abbreviations: TyG, triglyceride-glucose; CV, cardiovascular; eGFR, estimated glomerular filtration rate; MI, myocardial infarction; MACCEs, major adverse cardiovascular cerebral events; SHR, stress-hyperglycemia ratio; BMI, body mass index, T2DM, type 2 diabetes mellitus; HF, heart failure; AMI, acute myocardial infarction; DAPT, dual anti-platelet therapy.


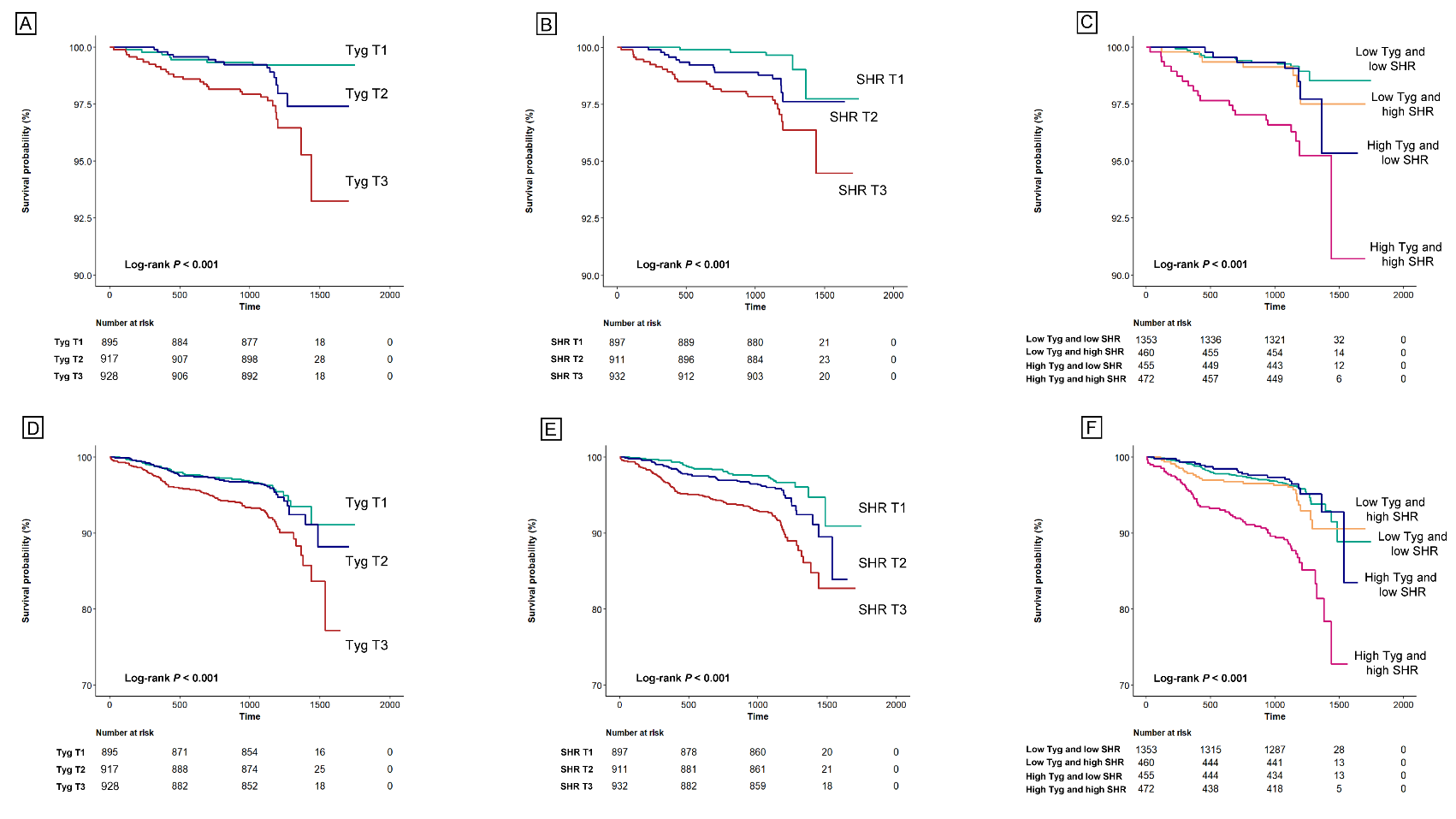


**Figure S1. Kaplan–Meier curves for the cumulative incidence of clinical outcomes in patients grouped by the TyG index (Figure S1A: CV death and TVMI; Figure S1D: MACCEs), SHR (Figure S1B: CV death and TVMI; Figure S1E: MACCEs), and combination of two ratios (Figure S1C: CV death and TVMI; Figure S1F: MACCEs).**

Abbreviations: TyG, triglyceride-glucose; CV, cardiovascular; TVMI, target vessel myocardial infarction; MACCEs, major adverse CV cerebral events; SHR, stress-hyperglycemia ratio.
